# Evaluating the Impact of the Level of Robustness in Operating Room Scheduling Problems

**DOI:** 10.3390/healthcare12202023

**Published:** 2024-10-11

**Authors:** Bernardetta Addis, Giuliana Carello, Elena Tanfani

**Affiliations:** 1 Université de Lorraine, CNRS, LORIA, F-54000 Nancy, France; bernardetta.addis@univ-lorraine.fr; 2 Department of Electronics, Politecnico di Milano, Information and Bioengineering, 20133 Milano, Italy; giuliana.carello@polimi.it; 3 Department of Economics, Università di Genova, 16126 Genova, Italy

**Keywords:** operating room planning, uncertain surgery duration, block scheduling, robust optimization

## Abstract

Managing uncertainty in surgery times presents a critical challenge in operating room (OR) scheduling, as it can have a significant impact on patient care and hospital efficiency. **Objectives:** By incorporating robustness into the decision-making process, we can provide a more reliable and adaptive solution compared to traditional deterministic approaches. **Materials and methods:** In this paper, we consider a cardinality-constrained robust optimization model for OR scheduling, addressing uncertain surgery durations. By accounting for patient waiting times, urgency levels and delay penalties in the objective function, our model aims to optimise patient-centred outcomes while ensuring operational resilience. However, to achieve an appropriate balance between resilience and robustness cost, the robustness level must be carefully tuned. In this paper, we conduct a comprehensive analysis of the model’s performance, assessing its sensitivity to robustness levels and its ability to handle different uncertainty scenarios. **Results:** Our results show significant improvements in patient outcomes, including reduced waiting times, fewer missed surgeries and improved prioritisation of urgent cases. Key contributions of this research include an evaluation of the representativeness and performance of the patient-centred objective function, a comprehensive analysis of the impact of robustness parameters on OR scheduling performance, and insights into the impact of different robustness levels. **Conclusions:** This research offers healthcare providers a pathway to increase operational efficiency, improve patient satisfaction, and mitigate the negative effects of uncertainty in OR scheduling.

## 1. Introduction

Hospital organisations worldwide are under increasing pressure to refine their healthcare delivery processes while simultaneously improving productivity and operational efficiency. As one of the most resource-intensive areas, surgical departments are critical to both the financial health of the institution and the quality of care provided. Not only do these departments contribute significantly to overall expenditure, but they also have a profound impact on patient outcomes, including service quality and waiting times. Due to the central role of surgical services, there has been a marked rise in research dedicated to enhancing Operating Room (OR) planning and management, aiming to streamline operations, reduce costs, and improve patient care.

Comprehensive reviews on OR planning and scheduling problems are reported in [1,2,3,4,5,6,7], where several variants of the problem and classification items are considered to guide researchers in correctly positioning their work. First of all, OR planning and scheduling problems are classified according to the considered scheduling policy. Scheduling policies may be roughly classified into block scheduling, open scheduling, and modified block scheduling. In block scheduling, each specialty schedules its surgical cases in a set of a priori determined OR blocks (usually half-day or full-day lengths) for the planning period [8]. In contrast, open scheduling allows specialties to share ORs and surgical cases can be assigned to any OR available [9]. The modified block scheduling strategy, which combines elements of both approaches, increases the flexibility of the pure block scheduling approach [10]. In this paper, we will assume a block scheduling strategy. Despite some potential negative impacts on the block schedule balancing [11] and overall OR utilization [12], this policy is currently widely accepted due to its convenience for surgical teams and managers [13] and its efficiency in exploiting surgeons’ time [14,15].

Within the block scheduling strategy, modeling and optimizing OR activities is usually divided into different problems which correspond to different decision levels [16]. At the strategic level, decisions are made about the number, type, and opening hours of ORs over a long period. At the tactical level, OR capacity is divided among the surgical groups or specialties that share the operating theatre facility. A cyclic timetable, known as the Master Surgical Schedule (MSS), is built to account for the tactical assignment of specialties to OR blocks over a medium-term planning horizon. Finally, at the operational level, surgery process scheduling deals with the scheduling and sequencing of surgical cases into the available OR blocks. In particular, in the advance scheduling phase a surgery date and an OR are assigned to each scheduled patient, and then, in the allocation scheduling phase, the sequence of surgeries in each OR block is determined.

In this paper, we will consider a block scheduling strategy and focus on the operational level of decision making with attention to the Advance Scheduling Problem (ASP). As in [17,18], we assume an operative scenario characterized by long elective waiting lists and extended waiting times, where not all patients on the waiting list can be scheduled within the considered planning horizon. Long waiting lists are a common feature of several healthcare systems, where patients are assigned to urgency classes based on their clinical conditions. Waiting lists for elective treatments and their associated waiting times are particularly important in the public sector and concerns surrounding their evaluation and control are receiving growing attention in the majority of OECD countries [19]. The problem addressed in this study involves selecting the subset of patients on a waiting list to be scheduled within the planning horizon, and determining their surgery dates and OR blocks. A patient-centered objective function is used, which accounts for waiting time, urgency, and tardiness. Given the significant impact of uncertainty in surgery times on hospital efficiency, this issue needs to be addressed and is therefore included in the problem. A robust solution is sought using a mathematical programming model based on the cardinality-constrained approach proposed in [20]. The contribution of this paper is a detailed study of the cardinality-constrained approach applied to the ASP, addressing the following questions:

(i) Is the aggregate objective able to provide good results from the single urgency class or the single patient’s perspective?
(ii) How can practitioners properly set the parameters of the method, and which analyses should be applied to help guide this choice?
(iii) We are forced to select approximate robustness parameters: how can appropriate robustness parameters be selected to provide good results in practice?
(iv) How do the selected robustness-related parameter values impact the behavior of the obtained solutions when applied in practice?
(v) Can the approach be applied to large waiting lists?

The behavior of the cardinality-constrained approach is investigated using a set of instances based on real data. According to a patient-centered perspective, the quality of the solutions with respect to the different patient classes is analyzed based on several metrics, such as the number of operated patients, patients still waiting, tardy patients, average waiting time, and tardiness. We perform a sensitivity analysis on the main robustness-related parameters, i.e., maximum surgery time deviation and robustness level. Their impact on the number of canceled patients and on the OR utilization rate, when applied to real scenarios, is evaluated. Finally, we investigate the scalability of the robust formulation of the problem applying the model to solve large-scale instances.

The remainder of the paper is organized as follows: in Section 2, we review the literature on similar problems. Section 3 formally introduces the problem, describes the modeling choices, and presents both the deterministic and robust formulations under investigation. In Section 4, the results are reported and discussed. Section 5 analyses the limitations of the work. Finally, in Section 6 conclusions are given.

## 2. Literature Review

Many researchers approached the ASP problem ignoring the stochastic aspects of surgery times and employing deterministic models to address the problem. Lagrangian relaxation approaches [21,22], branch and price and cut algorithms [23,24,25,26], heuristics [27,28,29,30] and metaheuristics algorithms [31,32,33,34] have been proposed to obtain reasonable solutions in acceptable computational times.

However, the problem is further complicated if the inherent uncertainty of surgery duration is accounted for, which forces planners to over-conservative scheduling, thus reducing the OR utilization level [35,36]. Modeling the variability of surgery times is a crucial factor and has a great impact on the resulting OR utilization and surgery cancellations [17,37,38]. Simulation based approaches have been widely used to compare alternative scheduling strategies and to assess the robustness of solutions in the face of unpredictable surgery durations [16,39,40], while the majority of studies have traditionally limited their focus to analyze and compare alternative scenarios, there has been a recent shift toward advanced simulation-based optimization approaches that integrate simulation with other solution techniques [41,42,43].

The optimization approaches that include the uncertainty within the ASP can be roughly classified into Stochastic Programming (SP), Robust Optimization (RO) and Distributionally Robust (DR) optimization methods [44].

In SP, the probability distribution of uncertain parameters needs to be perfectly known and the obtained solution protects against the most likely realizations. The high number of scenarios that must be generated to guarantee a good behavior of the method makes the resulting problem very challenging from the computational point of view. On the other hand, in DR, the probability distribution is assumed to belong to a family of distributions and the obtained solution protects against the worst-case scenario, thus leading to over-conservative solutions. Instead, RO approaches assume that each uncertain parameter belongs to a given convex set, so there is no need to generate scenarios nor to explicitly define the probability distribution, and it guarantees that the obtained solution is feasible for any parameter value within the considered uncertainty set.

In [45], a two-stage SP model with recourse is proposed and different heuristics are compared. The objective function includes the patient waiting times and the OR idle time and overtime. Furthermore, they analyzed the influence of patient sequencing inside the OR blocks. A stochastic programming model with recourse is also presented in [46] and a Sample Average Approximation (SAA) method is proposed to obtain surgery schedules that minimizes patient and OR overtime costs. In [47], a two-stage stochastic MIP model is proposed to determine the allocation of patients to OR blocks, the sequence of surgeries, and the starting time for each surgery. The model is used to quantify the impact of pooling ORs as a shared resource and of parallel surgery processing. This work aims at minimizing the total expected operating cost. Structural properties of the model are exploited in order to solve realistic-sized instances. Different heuristic approaches are proposed in [48], with the aim of maximizing the utilization rate of operating theater and minimizing the overtime risk by introducing slack times. A hybrid two-phase optimization algorithm to simultaneously address both the advance and allocation scheduling problem is introduced in [49], exploiting the potential of neighborhood search techniques combined with Monte Carlo simulation. The developed approach searches for a feasible and robust solution that strikes a balance between hospital efficiency and patient needs by maximizing OR utilization while minimizing patient cancellations. Similarly, Zhang et al. [50] propose a two-phase optimization framework that merges Markov decision processes with stochastic programming to enhance the long-term effectiveness of surgical scheduling. They further propose a column-generation-based heuristic algorithm, combined with a SAA method, to tackle realistically sized instances.

RO approaches have been proposed to account for the uncertainty of surgery durations and other aspects, such as the arrivals of emergent patients and the length of stay in ward and ICU beds, where the distributional information for the uncertain parameters is not known [51]. In [52], two models for minimizing the overall OR cost including a fixed cost of opening ORs and a variable overtime cost are compared. The first model is a two-stage stochastic linear model with binary decision variables in the first stage and simple recourse in the second stage. The second one is its robust counterpart, where the objective function minimizes the maximum cost associated with an uncertainty set for surgery duration. The robust method proved to be much faster than solving the stochastic recourse model, and is able to limit the worst-case outcome of the recourse problem. In [11], a chance constrained model is introduced to maximizes the expected utilization of ORs. The solution algorithm solves a series of MIP models based on a normal approximation of cumulative surgery duration. A two-level robust framework is proposed in [53]. In the first level, a MIP model finds a deterministic solution, while in the second level, the variability of surgery duration is taken into account by means of individual chance constraints for each OR block. The robust solution is achieved by iteratively adding safety slacks to the first level deterministic solution assuming lognormal distribution of the surgery durations. In [54], a mathematical program that represents uncertain duration of surgery procedures through probabilistic constraints is proposed. The model optimizes OR utilization without increasing overtime and cancellations.

Shehadeh and Padman [55] assume the probability distributions of random parameters to be ambiguous, and only the mean values and ranges are known. They propose a DR elective surgery scheduling model that minimizes the cost of performing and postponing surgeries and the worst-case expected costs associated with overtime and idle time of ORs also considering the ICU beds capacity.

In general, RO approaches are a good compromise between SP and DR optimization/ambiguous chance-constrained optimization. Among them, the cardinality-constraint approach proposed in [20] assumes that the uncertain parameters belong to an interval (the simplest convex set). To avoid over-conservative solutions, it does not guarantee to protect from the worst case scenario where each uncertain parameter assumes its boundary value, which is unlikely in real applications. Instead, given a parameter Γ, the solution is guaranteed to be still feasible if the elements in any subset of at most cardinality Γ assume their boundary value, and all the other parameters assume the value corresponding to the center of the interval. Therefore, the cardinality parameter Γ allows to tune the robustness of the method, and, if properly chosen, avoids to obtain over-conservative solutions. In summary, the cardinality-constrained robust optimization method combines a less challenging computational effort (not asking for scenario generation) with a good degree of flexibility that allows to adjust the trade-off between conservativeness/robustness and quality of obtained solutions. Last, but not least, the definition of a simple convex set (an interval) and a small number of parameters to be tuned (the cardinality parameter and the boundaries of the interval) make the cardinality-constrained approach easy to understand and easy to use for clinicians and health care service managers without any background in statistics or robust optimization.

The pros and cons of applying the cardinality-constrained robust approach in healthcare applications are thoroughly analyzed and discussed in [56].

This robust approach has shown promise in various healthcare management problems with uncertain data, such as home care service management [57] or bloodmobile routing [58]. In [18], a cardinality-constrained-based model was proposed for the ASP problem. However, the paper focuses on the impact of accepting overtime and does not analyse in depth the impact of the robustness level from the patients’ perspective, considering different urgency classes. In [17], a similar model is used within a rolling horizon approach. However, this paper also lacks an in-depth analysis of the impact of the robustness level.

In this direction, the present paper focuses on evaluating different levels of robustness and their impact on relevant metrics, taking into account both the hospital management and the patient’s perspective. In doing so, it bridges the gap between theoretical models and real-world applications. Such an evaluation is crucial for practitioners to effectively implement the approach in real-life scenarios according to their specific needs, analyzing the trade-off between robustness and solution quality in actual scenarios, which warrants further detailed discussion.

## 3. Methodology

As mentioned, we assume that the strategic and tactical decisions related to OR capacity planning and the master surgical schedule are given. We set our analysis at an operational level and we focus on the ASP related to a single surgical specialty. We assume a block scheduling strategy and an operative scenario characterized by long waiting lists, where the elective patients waiting for surgery are ranked according to a prioritization system based on five urgency classes [59]. For each urgency class a maximum waiting time (in days) is given, which represents the maximum number of days that a patient can wait before surgery without deteriorating their clinical conditions. The maximum waiting times are 8, 30, 60, 180 and 360 days, respectively. For each surgery type the statistical behavior of the surgery duration is known based on the clinical experience and on historical data. At the operational level, the surgical activities are usually planned on a weekly basis. Such short term does not allow to schedule all the waiting patients.

The problem can be defined as follows: selecting a subset of patients to be scheduled for surgery during the given planning horizon and assigning them to the available OR blocks, while guaranteeing that the capacity of each block is not exceeded. The problem focuses on a patient perspective, and aims at providing a good quality of service and patient satisfaction. The problem is solved with a cardinality constrained-based model which is described in the following section.

### 3.1. Mathematical Model

The planning horizon spans *D* days corresponding to one week in our tests. The set *J* of available OR blocks and their schedule during the week are given. The total available time of a OR block *j*, i.e., the block length, is denoted by $\gamma_{j}$. If a patient is scheduled in block j∈J, the number of days they wait is represented by a precomputed value $d_{j}$.

The patients in the waiting list at the beginning of the planning horizon are represented by the set *I*. Each patient *i* is characterized by a waited time $w_{i}$, that is the number of days they have already spent in the waiting list, and an uncertain surgery time ${\tilde{t}}_{i}$. Beside, each patient belongs to an urgency class. A maximum waiting time is associated with each class. Thus, the parameter $l_{i}$ represents the Maximum Waiting Time (MWT) of patient *i*, depending on the patient’s class. If the patient has spent $w_{i}$ days in the waiting list, they must receive surgery before the due date $l_{i}-w_{i}$, otherwise they are considered tardy.

A first modeling choice consists of defining an urgency parameter $u_{i}$ for each patient *i*. As introduced in [59], such parameters are used to weight in a different way the chronological time of the patients depending on the urgency class. The maximum waiting time contributes in defining the urgency coefficient $u_{i}$, which represents the speed at which the clinical need is assumed to increase with time. In particular, for each class the urgency coefficient is computed as the ratio between the maximum waiting time of the least urgent class and its own maximum waiting time.

In Table 1, we report a summary of the sets and parameters used in the model.

The patient-centered objective function must represent the quality of service provided to patients, which is improved by keeping the delay in meeting patients’ needs limited. In [28], an objective function has been proposed that minimizes the waited time of patients weighted by the urgency parameters. It takes into account waiting time of both scheduled and not scheduled patients. In [18], another term is added to the objective function that accounts for the due date violation and patient tardiness.

As second modeling choice, we consider an aggregate penalty function that accounts for both waited time and tardiness of all the patients, each weighted by the patient’s urgency parameter (terms $p_{ij}$). Further, the objective function accounts for unscheduled patients through a penalty ($q_{i}$):

$$\min\sum_{i\in I}\left\{\sum_{j\in J}p_{ij}x_{ij}+q_{i}\left(1-\sum_{j\in J}x_{ij}\right)\right\}$$

For these reasons, the objective function is the sum of two terms. The first term represents the penalty associated with scheduled patients. If patient *i* is scheduled in the block *j*, their penalty is:

$$p_{ij}=\left[d_{j}+{\left(w_{i}+d_{j}-l_{i}\right)}^{+}\right]u_{i}.$$

This term is composed of two parts: the number of days $d_{j}$ spent before receiving surgery from the beginning of the planning horizon and the tardiness ${\left(w_{i}+d_{j}-l_{i}\right)}^{+}=\max\left\{w_{i}+d_{j}-l_{i},0\right\}$, that is the number of days waited after the due date. The total number of days waited by patient *i* is $\left(w_{i}+d_{j}\right)$, however as the waited time $w_{i}$ cannot be reduced (as it is already passed), only the days $d_{j}$ waited in the planning horizon are penalized in $p_{ij}$. Since the waiting has not the same impact for patients of different urgency classes, the term is weighted for the urgency parameter $u_{i}$. We recall that $u_{i}$ is inversely proportional to $l_{i}$, therefore, one day spent for a patient of the most urgent class (their surgery must be performed in at most 8 days) counts as 45 days for a patient in the less urgent class ($l_{i}=360$).

The second term represents the penalty of the unscheduled patients. Let $q_{i}$ be the penalty parameter of patient *i* if they are not scheduled in any block:

$$q_{i}=\left[\left(w_{i}+D+1\right)+{\left(w_{i}+D+1-l_{i}\right)}^{+}\right]u_{i}.$$

It should account for the overall number of days spent before the surgery and for the tardiness. However, these quantities cannot be determined exactly in the current planning phase. Thus, as the third modeling choice, we note that the earliest day in which an unscheduled patient can receive surgery is D+1 and we compute the lower bound accordingly. The goal of this term is to guarantee that long waiting patients are not indefinitely delayed, rather than precisely computing their tardiness or waiting time. As for the scheduled patients, the waiting time and the tardiness are weighted by the urgency parameter $u_{i}$.

The problem can be formulated using binary variables $x_{ij}$, such that $x_{ij}=1$ if patient *i* is assigned to block j∈J, and zero otherwise. The Stochastic Advance Scheduling (SAS) model is formulated as follows:

(1) $$\begin{array}{cc} \left(SAS\right)\;\min\sum_{i\in I} & \left\{\sum_{j\in J}p_{ij}x_{ij}+q_{i}\left(1-\sum_{j\in J}x_{ij}\right)\right\} \end{array}$$

(2) $$\begin{array}{ccc} \; & \sum_{j\in J}x_{ij}\leq 1 & \;i\in I \end{array}$$

(3) $$\begin{array}{ccc} \; & \sum_{i\in I}{\tilde{t}}_{i}x_{ij}\leq \gamma_{j} & \;\forall j\in J,\; \end{array}$$

(4) $$\begin{array}{ccc} \; & x_{ij}\in \left\{0,1\right\} & \;\forall j\in J,\forall i\in I \end{array}$$

Constraints (2) ensure that each patient can be operated on at most once. Constraints (3) are the stochastic capacity constraints for each block forcing the total used time in block *j* to be less than or equal to the maximum available capacity time $\gamma_{j}$.

One quite common approach proposed in the literature to deal with uncertain parameters is to build a deterministic version of the model that accounts for the expected behavior of the parameters evaluating their expected values. For the considered problem the resulting Deterministic Advance Scheduling (DAS) model is the following, where ${\bar{t}}_{i}$ is the expected value of ${\tilde{t}}_{i}$:

(5) $$\begin{array}{cc} \left(DAS\right)\;\min\sum_{i\in I} & \left\{\sum_{j\in J}p_{ij}x_{ij}+q_{i}\left(1-\sum_{j\in J}x_{ij}\right)\right\} \end{array}$$

(6) $$\begin{array}{ccc} \; & \sum_{j\in J}x_{ij}\leq 1 & \;\forall i\in I \end{array}$$

(7) $$\begin{array}{ccc} \; & \sum_{i\in I}{\bar{t}}_{i}x_{ij}\leq \gamma_{j} & \;\forall j\in J,\; \end{array}$$

(8) $$\begin{array}{ccc} \; & x_{ij}\in \left\{0,1\right\} & \;\forall j\in J,\forall i\in I \end{array}$$

Other approaches, instead, aim at accounting also for other features of the uncertain parameters, such as their probabilistic distribution or a description of the uncertainty set. We resort to a robust counterpart of the DAS model to account for possible variations from the expected value. The robust model proposed in this paper is based on the robust optimization approach proposed in [20]: each uncertain parameter is assumed to have a reference value and a maximal allowed variation from that. A solution must be feasible even if, for each constraint involving uncertain parameters, at most Γ of them assume the maximum allowed value and all the others assume the reference one. In this case, the uncertain parameters are the surgery times ${\tilde{t}}_{i}$. The reference value of the interval is denoted as $\bar{t}$ and the maximum variation is denoted as ${\hat{t}}_{i}$.

For each block *j*, let $S_{j}\subset I$ be the subset of patients who require their maximum surgery time ${\bar{t}}_{i}+{\hat{t}}_{i}$. Only subsets of cardinality at most Γ are considered ($|S_{j}|\leq \Gamma$). Among all the possible subsets, the one having the worst impact on the capacity constraint is selected, and the solution is guaranteed to be feasible even with respect to such subset:

(9) $$\sum_{i\in I}{\bar{t}}_{i}x_{ij}+\max_{S_{j}\subset I:\left|S_{j}\right|=\Gamma }\left\{\sum_{i\in S_{j}}{\hat{t}}_{i}x_{ij}\right\}\leq \gamma_{j}\;\;\;\;\;\;\forall j\in J$$

Constraints (9) are not linear, but they can be linearized through duality (the complete procedure to derive the robust counterpart is reported in Appendix A.1), adding auxiliary variables $\pi_{i}^{j}$ and $\zeta^{j}$ such that:

(10) $$\begin{array}{ccc} \; & \sum_{i\in I}{\bar{t}}_{i}x_{ij}+\Gamma \zeta^{j}+\sum_{i\in I}\pi_{i}^{j}\leq \gamma_{j} & \;\forall j\in J \end{array}$$

(11) $$\begin{array}{ccc} \; & \zeta^{j}+\pi_{i}^{j}\geq {\hat{t}}_{i}x_{ij} & \;\forall j\in J,\forall i\in I \end{array}$$

(12) $$\begin{array}{ccc} \; & \zeta^{j},\pi_{i}^{j}\geq 0 & \;\forall j\in J,\forall i\in I \end{array}$$

We will refer to the robust model obtained replacing capacity constraints (3) with constraints (10)–(12) as the Robust Advance Scheduling (RAS) model:

(13) $$\begin{array}{cc} \left(RAS\right) & \min\sum_{i\in I}\left\{\sum_{j\in J}p_{ij}x_{ij}+q_{i}\left(1-\sum_{j\in J}x_{ij}\right)\right\} \end{array}$$

(14) $$\begin{array}{ccc} \; & \sum_{j\in J}x_{ij}\leq 1 & \;\forall i\in I \end{array}$$

(15) $$\begin{array}{ccc} \; & \sum_{i\in I}{\bar{t}}_{i}x_{ij}+\Gamma \zeta^{j}+\sum_{i\in I}\pi_{i}^{j}\leq \gamma_{j} & \;\forall j\in J \end{array}$$

(16) $$\begin{array}{ccc} \; & \zeta^{j}+\pi_{i}^{j}\geq {\hat{t}}_{i}x_{ij} & \;\forall j\in J,\forall i\in I \end{array}$$

(17) $$\begin{array}{ccc} \; & \zeta^{j},\pi_{i}^{j}\geq 0 & \;\forall j\in J,\forall i\in I \end{array}$$

(18) $$\begin{array}{ccc} \; & x_{ij}\in \left\{0,1\right\} & \;\forall j\in J,\forall i\in I \end{array}$$

For both parameters ${\bar{t}}_{i}$ and ${\hat{t}}_{i}$ a suitable value must be set before applying the robust approach, namely a value that meets the user requirement about the conservativeness of the solutions. We set ${\bar{t}}_{i}$ equal to the average of the uncertain distribution. This value allows a fair comparison between DAS and RAS. The choice of the maximum deviation ${\hat{t}}_{i}$ requires more analysis. In some applications, the distribution of uncertain parameters may not be bounded or the real maximum may be felt as too conservative by the user. In this case an alternative value must be selected for ${\hat{t}}_{i}$. However, the model is still meaningful and, by tuning the ${\hat{t}}_{i}$ value, it is able to meet the user willingness to risk ([56]). To provide some insights on how to select a suitable value for the OR planning case, we set the maximum deviation (the value we want to protect from) proportional to the standard deviation $\hat{t}=\alpha \sigma_{i}$. We analyzed the sensitivity to different values of α in Section 4.2.

### 3.2. Experimental Setting

DAS and RAS models have been implemented in AMPL and solved with CPLEX 12.2.0.0 on a Intel Xeon CPU E5335 (2 quad core CPUs at 2GH). We set a 2 h time limit and a 1 × $10^{-3}$ acceptable relative gap. The DAS and RAS models are tested on the set of instances described in Section 3.3.

To answer the research questions (i) to (v), introduced in Section 1, we perform different tests and analysis. The subjects of the analysis can be divided in three groups, each one reported in a different section:

- the effect of the objective function in selecting the patients to be operated on (Section 4.1),
- the effect of the choice of robustness parameters (Section 4.2),
- the computational time and scalability of the models (Section 4.4).

More in detail, in Section 4.1, we first investigate the impact of the aggregate objective function on patients belonging to different classes, setting Γ=4 and α=1, i.e., the maximum deviation is assumed to be equal to the standard deviation. Beside the objective function value itself, other metrics are measured, such as the number of operated and tardy patients for each urgency class, average waiting time and tardiness. For the waiting time, beside the absolute values, we also measure the weighted ones. In particular, for each patient we compute the Need-Adjusted-Waiting-Days (NAWD) that represents the waiting time adjusted by the urgency parameter [60]. The weighted waiting time expressed in NAWD can be used to measure the delay in meeting the clinical need of the patient.

Then we propose a set of computational tests meant to suggest how a practitioner should determine suitable values for robustness related parameters Γ and α (Section 4.2). Of course, such parameters should be evaluated before applying the models in practice. Therefore, we first suggest to analyze the results of the models to highlight the impact of different values of robustness parameters on patient based metrics and to determine a range of suitable values. Then, historical data and probabilistic description of the surgery times can be exploited to analyze the behavior of the solutions when applied to real life scenarios (scenario based analysis), so as to measure other important metrics such as OR utilization rate and number of canceled patients.

In Section 4.3, we discussed in detail a single instance example to show how the presented results can be used by practitioners to determine suitable values for the robustness parameters. Finally, computational time and scalability of the models are evaluated on a set of larger instances in Section 4.4.

### 3.3. Instance and Scenarios Generation

A time horizon *D* of 7 days (one week) is considered for all computational tests. For the computational results presented in Section 4.1 and Section 4.2, we used two sets of instances: one with 20 patients in the waiting list and two OR blocks available per week, and another with 40 patients and three OR blocks per week. Each block *j* is assumed to have a 6-h capacity. The patient characteristics for each instance are generated using real waiting list data collected from a surgical department of the San Martino Public Hospital in Genoa, Italy, for a one year period. Each waiting list gives the set *I* of patients who are waiting for surgery and should be scheduled; for each patient *i*, we get the date of referral and corresponding waiting time $w_{i}$ and the urgency class and corresponding urgency status $u_{i}$.

The initial situation of the 20 and 40 patients instances is given in Table 2. The following values are reported for each Urgency Related Group (URG), from the more urgent (0) to the less urgent (4), and for the overall waiting list:

- the Maximum Waiting Time (MWT) –$l_{i}$ in the model formulations–,
- the number of patients (p),
- the number of tardy patients (t_p) at the beginning of the planning horizon,
- the fraction of tardy patients with respect to the total number of patients at the beginning of the planning horizon (t_p/p),
- the average waiting time (wt) and the average weighted waiting time (wwt) of all patients,
- the average tardiness (trd) of tardy patients.

As previously mentioned, the weighted waiting time (wwt) is expressed in NAWD: the waiting time of each patient is multiplied for the urgency coefficient of the urgency class to which the patient belongs. The overall value of wt, wwt and trd are computed as weighted averages (with respect to the number of patients in each urgency class) of the overall list *I*. These values measure the difficulty of handling the initial list and its clinical need. They represent, respectively, the total priority score, i.e., total clinical need of the waiting list expressed in NAWD and the overall tardiness of all the patients waiting at the beginning of the planning horizon.

Urgency classes 1 and 2 contain the majority of the tardy patients. In fact, the average waiting time for the patients in such classes is higher than the maximum allowable waiting time and the average tardiness is quite significant. For the *I* = 40 patients waiting list the average tardiness of patients in urgency class 1 is 37 days: this means that tardy patients have been waiting for more than twice their maximum allowed waiting time, i.e., 30 days.

Surgery times were derived from [48]. We used data coming from eight different sets of surgery times. Each set consists of different types of surgeries and each type of surgery is characterized by an average surgery time, a standard deviation and the percentage of that type of surgery in the total number of surgeries. Given a waiting list and a surgery times set, we assigned to each patient *i* a surgery type, along with the associated average surgery time (${\bar{t}}_{i}$) and standard deviation ($\sigma_{i}$). Thus, for each waiting list, we generated eight instances by assigning to the patients the different surgery times coming from the various surgery sets. Each generated instance is named n−s, where *n* is the the number of patients in the waiting list (|I|), and *s* is the surgery time set.

To evaluate OR utilization and patient cancellations at various robustness levels, when applied in practice, we generated 100 random realizations for each instance. In each realization, the surgery time ${\tilde{t}}_{i}$ for each patient *i* was randomly generated using a lognormal distribution with an average surgery time ${\bar{t}}_{i}$ and a standard deviation $\sigma_{i}$, as recommended in previous studies [61,62,63,64,65]. To prevent unrealistically short surgery times, we truncated the distribution at a minimum value equal to $\max({\bar{t}}_{i}-\sigma_{i},30)$. If $r_{i}$ is the randomly generated value from the lognormal distribution, the surgery time assigned to patient *i* is set as ${\tilde{t}}_{i}=\max\left(r_{i},{\bar{t}}_{i}-\sigma_{i},30\right)$.

Additionally, we assessed the scalability of the method on larger instances (Section 4.4) by considering three waiting lists with 80, 120, and 140 patients, respectively. These larger instances were generated using the same procedure as the smaller ones. For each waiting list, two sets of instances were generated using the same procedure as the smaller one: one with three OR blocks per week and another with five blocks per week.

## 4. Results and Discussion

We carried out three types of analysis on the behaviour of the models. First, we analyse the impact of the aggregated objective function, so as to answer to the first research question, as described in the introduction (Section 1). Then, we evaluate the impact of the level of robustness, so as to answer to research questions (ii) to (iv). Finally, we tested the models on large instance, so as to answer to the last research question (v).

### 4.1. Analysis of the Objective Function and Impact on Urgency Classes

In this Section, the results produced by the aggregate objective function, both for the DAS and for the RAS models, are analyzed focusing on their impact on different urgency classes. The quality of the obtained solutions is evaluated based on the following metrics:

(a) Table 3:
  - the number of operated (op) and still waiting (sw) patients at the end of the planning horizon,
  - the number of tardy patients, either operated (op_t) or still waiting (sw_t),
  - the fraction of tardy patients with respect to the total number of patients at the beginning of the planning horizon (t_p/p)
(b) Table 4:
  - the average waiting time for the operated (wt_op) and the still waiting patients (wt_sw),
  - the average weighted waiting time (wwt) of the operated (wwt_op) and the still waiting patients (wwt_sw), expressed in NAWDs,
  - the average tardiness both for the operated (trd_op) and the still waiting patients (trd_sw).

We set Γ=4 and α=1 in this analysis. In Table 3 and Table 4, average results with respect to different operating time sets are reported for the 20 and 40 patients instances, a “-” is used where the value was not available/applicable (for example, when there are no operated patient associated with a class, then the corresponding metrics are marked by “-”). All the metrics are reported both as average values for each urgency class and for all instances (weighted average with respect to the number of patients in each urgency class, lines marked as *overall*). For additional information, in Table A1 and Table A2 (Appendix A.2), the detailed results for each operating time set are reported.

In general, the solutions select more patients from urgency classes 0 to 2—the most urgent—while patients in urgency classes 3 and 4 are seldom operated on (Table 3). Looking at the detailed results (Table A1 and Table A2): for the 20 patients list, one patient in one list (20−4) for the DAS model (none for the RAS), for the 40 patients instances, one or two patients in three lists for the DAS model (40−3, 40−4, 40−8), and in one list for the RAS model (40−5).

To analyze the effect of the objective function, it is interesting to compare the results of the two models (DAS and RAS) with respect to the initial situation of the waiting list reported in Table 2. The number of tardy patients at the end of the planning horizon (sw_t) is always reduced with respect to the initial situation, although the improvement is less significant when robustness is required, namely for the RAS model. In fact, applying the DAS model, the overall percentage of tardy patients at the end of the planning horizon (t_p/p) is reduced with respect to the initial situation of 6 percentage points for the 20 instances (passing from 40% to 34%) and of 14 percentage points for the 40 instances (passing from 25% to 11%). The average reduction drops to 1 and 9 percentage points for the 20 and 40 instances, respectively, applying the RAS model. The most significant reduction is shown in urgency classes 1-3 where the highest number of tardy patients was concentrated at the beginning of the planning horizon. For example, for urgency class 1 the reduction of tardy patients reaches the 14 and 13 percentage points for the 20 instances (from 50 to 36 and 37), respectively, applying the DAS and RAS model, and 32 and 28 percentage points for the 40 instances (from 50 to 18 and 22).

For all instances, not only the number of tardy patients, but also the average tardiness of patients still waiting at the end of the planning horizon (trd_sw, Table 4) is reduced with respect to the initial situation. The improvement for the 20 instances is about 36% and 35%, in the DAS and in the RAS model, respectively. Starting from 35.25, it drops to 22.64 for the DAS model and 22.95 for the RAS model. The improvement is more significant for the 40 instances where it reaches about 49% and 46%, in the DAS and in the RAS model, respectively. Starting from 27.6, and dropping to 14.03 (DAS) and 14.96 (RAS).

A reduction of the average tardiness is obtained both for the DAS model and the RAS model, although requiring robustness increases the average tardiness at the end of the planning horizon. The average tardiness is very sensitive to the distribution of the surgery times. For instance, it may vary from 2 up to 18.60 for the 40 patients case applying the DAS model, from 2 up to 21 for the 40 patients case applying the RAS model (see Table A2).

The reduction of the average tardiness of still waiting patients is due to the fact that scheduled (operated) patients are mainly chosen among the patients who have a greater need. In fact, the tardiness of the operated patients (trd_op) is always above the average initial tardiness, which considers all the patients in the waiting list at the beginning of the planning period. On the other hand, the average waiting time of patients in the waiting list in general increases with respect to the initial value (this increase is less important in the 40 patients instances). In general, this average increase is due to an increase for the less urgent (and often not tardy) patients, to allow a reduction on the most urgent classes (where there are on average less tardy patients, due to the largest MWT). This is a direct consequence of giving priority to most urgent classes and gives as result that the average waiting time for not operated patients (wt_sw) stays or drops below the MWT for almost all the different urgency classes instances and for both models.

The overall wwt_op and wwt_sw (expressed in NAWD) quantifies, respectively, the amount of the met need, that is, the need satisfied during the period, and of the still unmet need. If robustness is required the met need decreases and the unmet need increases. For all the instances (20 and 40) the average improvement of the overall weighted waiting time (wwt_sw) with respect to the initial situation (see Table 2) is on average 13% and 9%, respectively, for the DAS model and the RAS model.

Note that in these tests new patient arrivals are not taken into account. Indeed, new patients cannot become tardy patients during the planning horizon (even for the most urgent class), and thus do not affect the overall tardiness values and mainly reduce the average waiting time.

### 4.2. Evaluating the Impact of the Level of Robustness

As mentioned, practitioners must set the value of the robustness related parameters when applying the cardinality constrained robust approach to the health care problem under consideration. The choice of such values depends on the willingness to risk or, conversely, on the level of robustness required by the practitioners together with the features of the uncertain parameters. Thus, users have to evaluate the impact of different choices of such values on some relevant metrics and, based on this analysis, select the most suitable values for their care settings.

For the considered OR planning problem, the practitioners have to set the values of Γ and α. Several metrics should be considered. Some of them are related to the quality of service provided to patients, such as waiting time and number of cancellations, while others represent quality measures related to the hospital point of view, such as OR utilization rate.

In this section, we provide a set of analysis and results whose aim is to evaluate the impact of robustness parameters on relevant performance metrics. We present two sets of results. The first set shows the outcomes of the models, while the second set represents the behavior of the obtained solutions in practice, namely when the solutions are applied to a set of scenarios representing real life situations. The analysis on the ex-post realizations is very important as it shows the impact of the choice of the robustness parameters on the behavior of the solutions whereas they will be applied in practice.

#### 4.2.1. Analysis of the Obtained Solution

First, we analyze the impact of different values of parameters Γ and α, that is to say, of different choices of the maximum deviation, on the quality of solutions of the robust model (RAS). We run the model with $\alpha \in \left\{0.5,1,2\right\}$ and $\Gamma \in \left\{1..8\right\}$.

**Figure 1:**
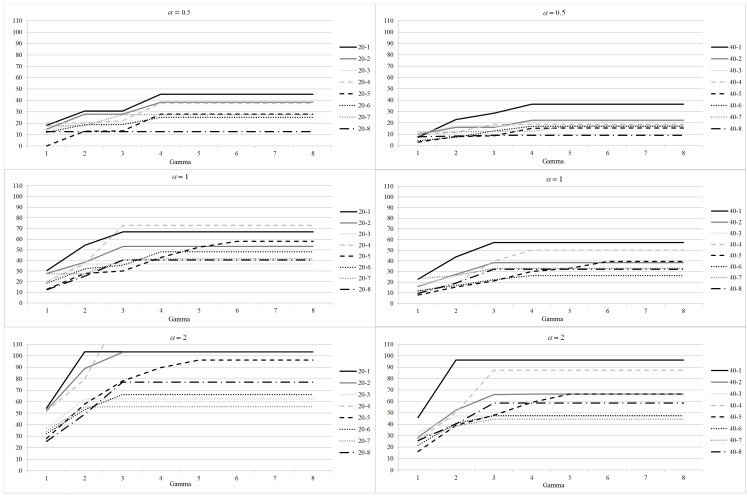
Robustness parameter analysis: average increase per patient of the objective function with respect to the DAS model for different values of α.

All the metrics are reported not in absolute value, but as the increase with respect to the deterministic model (DAS) to highlight the impact of robustness. In Figure 1, we consider the increase in the objective function with respect to the non robust case (DAS model) for 20 and 40 patients instances. Results for the 20 patients instances are reported on the left, while results for the 40 patients instances are given on the right. For all the considered values of α and Γ, we plot the average increase per patient expressed in NAWD. We recall that the value expressed in NAWD quantifies the priority score of a patient operated or still waiting at the end of the planning horizon: for instance, let us consider the case with 20 patients and α=2 (third figure on the left). We observe that for the list 20−1 and Γ=2 the waiting time per patient is increased of 100 NAWD with respect to the case where no robustness is required (DAS model).

This means that for a patient in the less urgent class (class 4, l=360, u=1) the waiting time is increased on average of 100 days, while for a patient in the most urgent class (class 0, l=8, u=45) it is increased of 2.2 days (100/u). That is, for each patient, passing from the non robust case to the robust case, the average waiting time is increased of 28% with respect to their maximum waiting time.

Results show that the impact of α and Γ combine, as they both increase the total surgery time required by a set of patients assigned to the same block. For a given value of Γ, increasing the value of α increases the average delay. In fact, the choice of the α value is linearly correlated with the length of the maximum surgery duration ($\bar{t}+\alpha \sigma_{i}$), thus increasing the value of α results in reducing the number of patients operated per block. Analogously, for a given value of α, increasing Γ requires a greater number of patients to require their maximum surgery time. However, the increase of the objective function is constant after a value $\underline{\Gamma }$ which depends on the value of α and varies for the different instances considered. In fact, if the value of Γ is equal to, or greater than, the maximum number of patients who can be scheduled in a given block, all such patients are assumed to require their maximum surgery time. Such value in general decreases with the increasing value of α. Let us consider, as an example, instance 20−1. If α=2 the maximum increase is reached for Γ=2, and it is slightly more than 100 NAWD. Such value is never reached if α=1 or α=0.5. For α=1 the maximum is between 60 and 70 NAWD, and it is reached for Γ=3. Instead, for α=0.5 the maximum is less than one half of that obtained for α=2, and it is reached for Γ=4. As a general remark, we note that, for a given value of α, increasing the value of Γ above $\underline{\Gamma }$ is thus useless.

In order to analyze in detail the impact of Γ, let us select α=1 (second line of plots in Figure 1).

The distribution of the surgery times has a significant impact on the increase of the waiting time. As an example, for instance 20−4, the value almost doubles the value for instance 20−8 for Γ≥3. Besides, the impact of the required level of robustness is different for the different instances, as well. Instance 20−4 seems to be affected the most by the increasing value of Γ: the average increase of the waiting time is about 20 NAWD for Γ=1 but, rises up to more than 70 NAWD for Γ≥3. On the other hand, the increase of the average waiting time is smaller for instance 20−7, as it is almost 30 NAWD if Γ=1 and rises up to 40 NAWD for higher values of Γ. For the 40 patients instances, the increase with respect to the deterministic case is not so high: in general it is below 30 NAWD for Γ=1 and never rises above 60 NAWD even for Γ=8. In general, the behavior is different for different instances and may vary significantly (see, e.g., the behavior of instances 40−1 and 40−6). The surgery times impact in a slightly dissimilar way when combined with 20 or 40 patients lists, as shown by instances 20−1 and 20−4 and 40−1 and 40−4.

Beside the increase of the objective function, let us consider other metrics: the number of operated patients and the number of tardy patients, either operated in the considered time horizon or still waiting at the end. It is worth pointing out that such metrics are not explicitly included in the objective function of the optimization models, as the latter accounts for the sum of the weighted delay and tardiness of all the patients.

Table 5 presents the number of patients operated on for each instance and for each value of Γ. The first column (max) provides an upper bound on the number of patients that can be operated on, serving as a benchmark. This upper bound is obtained solving a knapsack-like problem that maximizes the number of operated patients without considering patient penalties. As previously mentioned, the number of operated patients remains constant for $\Gamma \geq \underline{\Gamma }$, and this value is therefore indicated by a “-” in the table.

The results indicate that when robustness is not a requirement (as in the DAS model), the number of operated patients approaches the upper bound (max). As the value of Γ increases, there is typically a decrease in the number of operated patients. However, for low values of Γ, the number of operated patients remains very close to the upper bound. Even with higher Γ values, the number of operated patients rarely falls below half of the maximum benchmark; in most cases, it remains above 50% of this bound. It is important to note that the set of patients operated on varies with different values of Γ. In general, the set of patients operated on for Γ=m is not necessarily a subset of those operated on for Γ=m−1, indicating that changes in Γ result in a different selection of patients, rather than just a reduction in the number of patients operated on. This highlights the complexity of the trade-offs involved in adjusting robustness requirements. This can be verified considering the patients more in details. Let us focus on tardy patients, namely those patients whose deadline has been exceeded at the end of the planning horizon, who represent a criticality of the system with respect to the quality of service provided to patients. In Table 6, the number of patients who receive surgery after their due date is reported in the “op” columns, while the number of tardy patients still waiting at the end of the planning horizon is given in the “sw” columns. As the objective function takes into account the weighted waiting times, rather than the number of tardy patients, their number may increase or decrease when Γ increases. In general, the differences are not dramatic. In most of the instances with 20 patients the number of still waiting tardy patients increases of one or two units when a high level of robustness is required. The greatest increasing of the number of tardy patients still waiting is experienced for instance 20−4, where such number increases from 2 to 5. The increasing is higher for the 40 patients instances, where it is below 2 for 5 instances and rises up to 4 for instance 40−4.

We recall that, at the beginning of the planning horizon, 20 patient instances already have 8 tardy patients, while 40 patient instances have 10 tardy patients (see Table 2). Such numbers are always reduced at the end of the planning horizon. However, the final number significantly depends on the surgery times. In particular, the number of tardy patients is significantly reduced for surgery sets 4 and 5, especially for low level of robustness (e.g., all the tardy patients are operated on in instances 40-4 for DAS model). The reduction is less significant for higher values of Γ.

#### 4.2.2. Scenario Based Analysis

If we focus only on metrics related to model outcomes, robustness might seem to have only the negative effect of reducing the number of operated patients and increasing the patients’ delay. Therefore, it is necessary to assess what would happen in the real system if the model solutions were applied. In fact the benefits of a robust solution become evident when dealing with unpredictable and unexpected variations in the surgery times. We tested the solutions obtained by the models for different values of α and Γ on a set of scenarios representing realizations of patient surgery times, so as to evaluate the metrics on which robustness has a positive impact, such as disruptions caused by canceled patients. The surgery time realizations are generated as described in Section 3.3. The obtained solutions are evaluated with respect to two meaningful metrics, i.e., the utilization rate of the operating rooms and the number of canceled patients, representing, respectively, the hospital management perspective and the patient one. Canceled patients depend on the realization of surgery times, but also on hospital policies. To obtain an estimation of number of canceled patients, we used the following working hypothesis: in each block, surgeries are scheduled according to the Longest Processing Time (LPT) rule based on average surgery time (i.e., from the largest to the smallest surgery time). Patients are considered one by one according to the LPT scheduling. If there is still some residual time, the patient is operated on and the residual time is reduced by their realized surgery time. Otherwise the patient is considered canceled and so the remaining patients in the LPT list.

**Figure 2:**
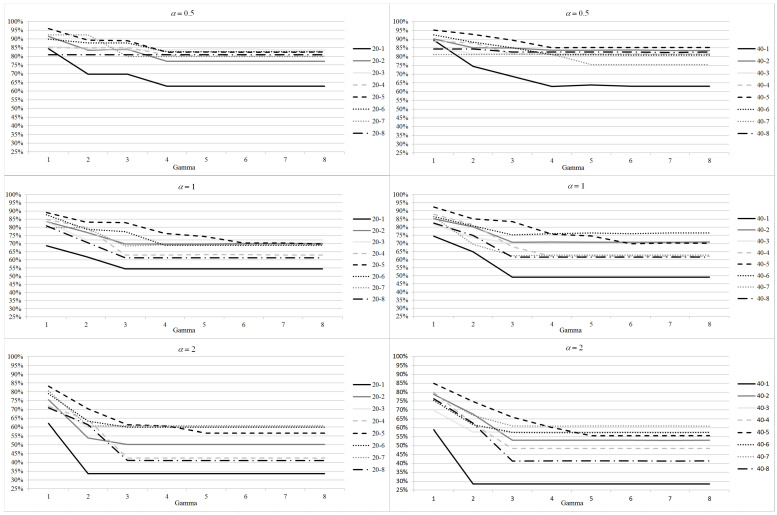
Robustness parameter analysis: increase in utilization rate with respect to DAS model for different values of Γ and α.

**Figure 3:**
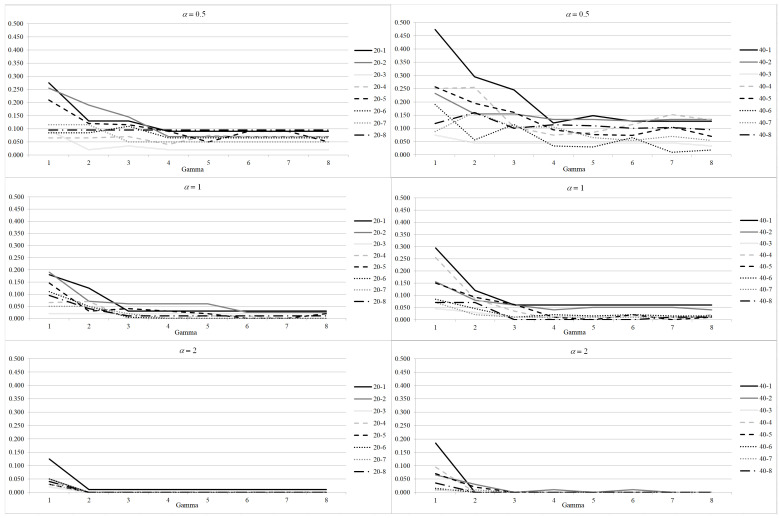
Robustness parameter analysis: increase of average number of canceled patients per block with respect to DAS model for different values of Γ and α.

Results on the average utilization rate and on the number of canceled patients per block are shown in Figure 2 and Figure 3, respectively. The results are averaged out on the 100 scenario realizations. As shown for the increase of the objective function, the impacts of parameters α and Γ combine. The utilization rate decreases with the increasing value of α and Γ. In fact, when a higher level of robustness is required, a higher amount of operating room resources must be reserved to deal with the increase of the surgery times the users want to protect from. For the 20 patients instances, when α=0.5 and Γ=1, the utilization rate is high, being between 80% and 95% for all the instances. Such value decreases when Γ increases: it is between 70% and 90% when Γ=4, and it drops between 60% and 85% for the highest value of Γ. Utilization rate is lower for α=1, as it never rises above 90%, not even for Γ=1. Indeed it is below 70% for all the instances when high levels of robustness are required. The utilization rate is even smaller for α=2. It never rises above 85%, and for instance 20−1 it drops below 35% even for Γ=2. A somehow similar behavior can be noticed for 40 patients instances. So requiring high level of robustness, increasing Γ or α, may produce low quality solutions from the hospital management perspective. However, the benefit of robust solutions are evident when the number of canceled patients is analyzed.

In fact, increasing the level of required robustness reduces significantly the number of canceled patients thus reducing disruptions and increasing the quality of service perceived by patients. When α=0.5 the number of canceled patients is below 0.1 for the 20 patients instances and below 0.15 for the 40 patients instances when Γ≥4. The impacts of Γ and α combine in reducing the number of canceled patients. If α=1, even Γ=3 is enough to guarantee a number of canceled patients below 0.6. For α=2 the number of canceled patients is almost 0 even for Γ=2. A similar value cannot be achieved for lower values of α even for very high values of Γ.

### 4.3. Discussion of a Detailed Example

**Figure 4:**
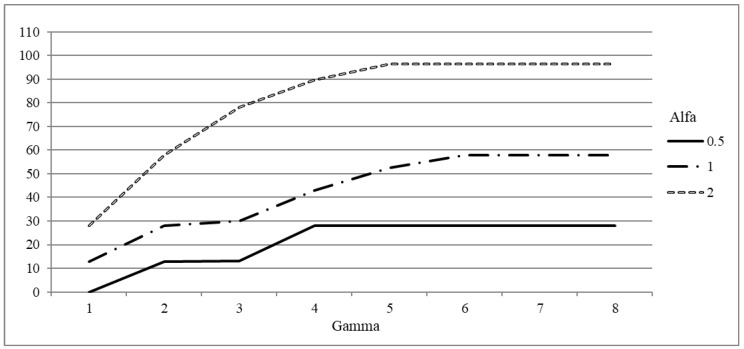
Average per patient objective function increase for different values of Γ and α (instance 20−5).

**Figure 5:**
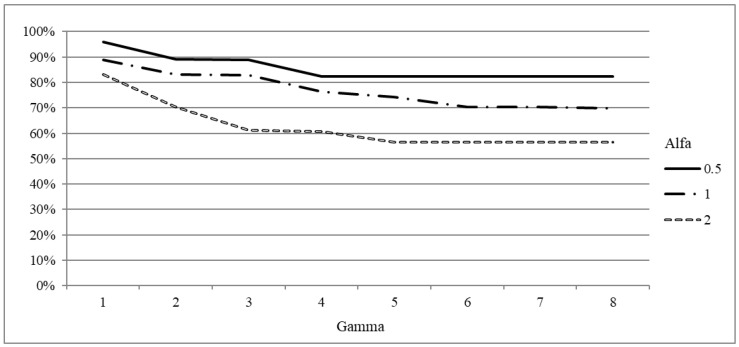
Average utilization rate for different values of Γ and α (instance 20−5).

**Figure 6:**
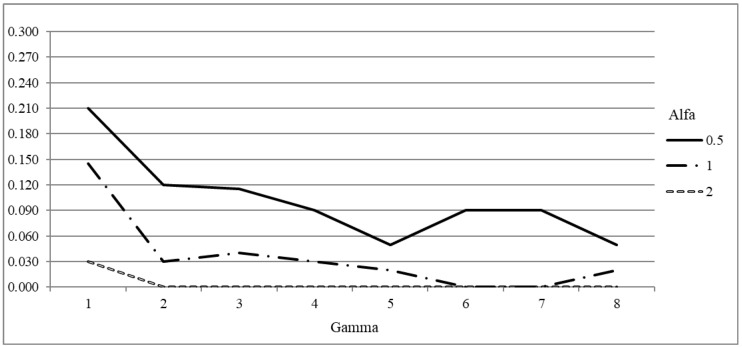
Average number of canceled patients per block for different values of Γ and α (instance 20−5).

We now focus on a single instance and surgery time set, namely instance 20−5, to show how the presented results can be used by practitioners to determine suitable values for the robustness parameters. In Figure 4, Figure 5 and Figure 6, the average per patient increase of the objective function, the utilization rate and number of canceled patients per block for the scenario based analysis are reported, respectively.

The figures highlight the impact of choosing different levels of robustness and the way in which their impact combines. Let us considered for instance the average increase of the objective function for Γ=1 and Γ=2 (Figure 4). A delay of about 30 NAWD is obtained either for Γ=1 and α=2, or for Γ=2 and α=1. A value slightly below 60 NAWD is obtained either for Γ=2 and α=2, or for Γ=6 and α=1. Thus, the impacts of the two parameters are combined, but a high value of α produces behaviors that cannot be obtained for lower values of α just by increasing Γ.

Concerning the number of canceled patients, a null value can be obtained for any Γ≥2 if α=2. Instead if α=1 we must set Γ=6 to obtain no cancellations and we cannot reset the number of cancellations if α=0.5, whatever value of Γ is selected. Therefore, if the practitioner asks for no cancellation at all, Γ=2 and α=2 are suitable values, but the price to pay is twofold: an increase of the patient delay of 60 NAWD (around 17% with respect to the deterministic case), and an OR occupation of 70%. In fact about one third of the OR available time is left unused to face unpredictable increase, so as to have enough operating room capacity to operate all the scheduled patients. On the other hand, if some cancellations are accepted (0.05 cancellations per block, that is on average a cancellation every 7 weeks), α can be set to 1 and Γ to 2, thus reducing the delay of one half (30 NAWD) and increasing the room occupation up to 85%.

The values of Γ and α must be carefully tuned to evenly balance the utilization rate and the number of canceled patients, and to produce schedules that satisfy the requirements of both patients and hospital management stakeholders. Increasing α and Γ have a twofold impact: on the one hand it reduces the number of operated patients, thus increasing the waiting time, on the other hand, it reduces the disruptions, thus improving the quality of service and the hospital organization. In fact, canceled patients must be rescheduled in the weeks after the current planning scenario, therefore they have a strong impact not only on the quality of service perceived by patients but also on the future OR planning.

### 4.4. Scalability of the Method

The last set of tests aims at evaluating the scalability of the proposed models when applied to larger instances. In Table 7, the average and maximum computational times are given, for the DAS and RAS models, for the 20 and 40 patients instances, for each value of Γ and for α=1. All the considered instances with 20 or 40 patients are solved to optimality within the two hours time limit. Solving the deterministic model requires few seconds, while the computational time may significantly vary for the robust counterpart. Computational time is higher for higher values of Γ, with a peak corresponding to Γ=5 or Γ=6. However, the required CPU time is never above half an hour.

In this section, we consider three additional sets of instances with 80, 120 and 140 patients, respectively, and three and five blocks, in order to evaluate the models scalability with respect to the number of patients and blocks. Table 8 shows the CPU time and percentage gap for large instances, averaged over all the surgery time sets, while Table A3, Table A4 and Table A5 give detailed values for the 80, 120 and 140 patient instances, respectively. The gap is denoted with “ag” when the solver manages to reach the acceptable gap of $1\times 10^{-3}$. For instances where the relative acceptable gap is not achieved within the two-hour time limit, the computational time is denoted as “TL”. Instances that run out of memory are marked with “*”. Additionally, average and maximum computational times are reported for each instance.

The DAS model is solved to optimality for all the instances with 80 patients (Table A3). Computational times are small for the scenario with three OR blocks and increase moderately for the five-block scenario. However, even with five blocks, the average computational time is around 10 s, exceeding one minute in only one instance. For the RAS model, five instances with 80 patients and three blocks were not solved to optimality. As the value of Γ increases, so does the computational effort required. For instances solved to optimality, the average CPU time rises with increasing Γ values, peaking at Γ=6 and Γ=7, before slightly decreasing for Γ=7 and Γ=8. This pattern may be due to the fact that, at higher Γ values, nearly all scheduled patients require their maximum surgery times. The computational time of the RAS model varies significantly across different surgery time sets, with instances 80−4 and 80−5 proving to be the most challenging. For instances not solved to optimality, the gap remains below 1.5%. The number of blocks has a significant impact on computational time, with both the average CPU time and the number of unsolved instances increasing significantly. In addition, seven instances ran out of memory.

For the instances with 120 patients (Table A4), the DAS model also solves all cases to optimality within a limited computational time. Looking at the RAS model results, increasing the number of patients from 80 to 120 results in a noticeable increase in computational time, though this impact is less pronounced than the effect of adding two additional blocks. All instances with 120 patients and three blocks are solved to optimality except seven, which still show a very low gap. Conversely, almost all instances with five blocks cannot be solved to optimality, with three of them running out of memory. Nonetheless, the gaps in these cases are reasonable, averaging between 0.34% and 2.67%, and never exceeding 6.5%. This suggests that while the RAS model becomes more challenging as the instance size increases, the solutions remain fairly close to optimal, even in more complex scenarios.

A similar pattern is observed in the 140 patient instances (Table A5), although one instance runs out of memory even when solving the DAS model.

## 5. Limitations

The proposed approach focuses solely on a single operating room (OR) and does not consider surgeon constraints, or bed availability. While this simplified setting allows for a thorough analysis of the proposed method, it limits the reliability of the numerical results. A new analysis will be necessary to quantify the performances when the method is applied to more complex OR scheduling scenarios. Furthermore, while we have incorporated uncertainty in surgery times, our model does not account for other potential sources of uncertainty, such as no-shows or emergent urgent cases. This limitation restricts the scalability of our approach to more complex problems, as the computational burden may increase significantly when considering additional uncertainties. Despite these limitations, our research provides a solid foundation for future work. Heuristic strategies can be explored to address the challenges posed by multiple ORs, surgeon constraints, and bed availability. Additionally, incorporating additional sources of uncertainty into the model can enhance its realism and applicability to a wider range of OR scheduling scenarios.

## 6. Conclusions

This paper presents a cardinality-constrained robust optimization approach for OR scheduling, addressing challenges posed by long waiting lists, varying urgency classes, and uncertain surgery durations.

A key contribution of this work is the comprehensive analysis of the proposed approach’s performance and sensitivity to key parameters. We demonstrate the effectiveness of the aggregate objective function in balancing the needs of scheduled and unscheduled patients, focusing on prioritizing urgent cases and minimizing tardiness.

Our analysis of the robustness parameters provides valuable insights for practitioners. While robustness can lead to some performance degradation in offline evaluations, it offers significant benefits in real-world scenarios, effectively mitigating disruptions and ensuring a high level of service. By carefully tuning these robustness parameters, healthcare providers can achieve an optimal balance between operational efficiency and patient satisfaction. Furthermore, we established the computational feasibility of the proposed approach. The computational time required for solving the model is compatible with real-world scheduling requirements, even for large-scale instances. This makes our approach a practical tool for OR scheduling in healthcare settings.

In conclusion, this research offers a robust and efficient solution for OR scheduling problems. The insights gained from our analysis can be used by healthcare practitioners to optimize their scheduling processes, improve patient outcomes, and enhance overall hospital performance.

## Figures and Tables

**Table 1:** Summary of the model parameters.

Name	Description
*I*	set of patients in the waiting list
*J*	set of OR blocks available in a week
${\tilde{t}}_{i}$	surgery time of patient *i*
$w_{i}$	waiting time of patient *i* at the beginning of the planning horizon
$l_{i}$	maximum waiting time of patient *i*
$u_{i}$	urgency parameter of patient *i*
$\gamma_{j}$	capacity of block *j*
$d_{j}$	days waited until block *j*
*D*	total number of days in the planning horizon

**Table 2:** Initial situation of the 20 and 40 patients waiting lists.

Instance	URG	MWT	p	t_p	t_p/p	wt	wwt	trd
*I* = 20	0	8	1	0	0.00	6.00	270.00	-
	1	30	6	3	0.50	34.00	408.00	24.67
	2	60	8	3	0.38	66.75	400.50	52.00
	3	180	4	2	0.50	173.75	347.50	26.00
	4	360	1	0	0.00	338.00	338.00	-
	overall	-	20	8	0.40	88.85	382.50	35.25
*I* = 40	0	8	1	1	1.00	12.00	540.00	4.00
	1	30	8	4	0.50	38.88	466.56	37.00
	2	60	26	4	0.15	44.04	264.24	27.00
	3	180	3	1	0.33	158.33	316.66	16.00
	4	360	2	0	0.00	334.50	334.50	-
	overall	-	40	10	0.25	65.30	319.04	27.60

**Table 3:** Impact of the objective function: number of (#) operated and still waiting patients (total and only tardy patients).

		DAS					RAS (Γ=4)				
		# Patients		# Tardy p.			# Patients		# Tardy p.		
Instance	URG	op	sw	op_t	sw_t	t_p/p	op	sw	op_t	sw_t	t_p/p
*I* = 20	0	0.75	0.25	0.00	0.25	1.00	0.63	0.38	0.00	0.38	1.00
	1	2.50	3.50	1.75	1.25	0.36	1.63	4.38	1.38	1.63	0.37
	2	4.50	3.50	3.63	0.75	0.21	3.50	4.50	3.38	1.50	0.33
	3	0.13	3.88	0.13	1.88	0.48	0.00	4.00	0.00	2.00	0.50
	4	0.00	1.00	0.00	0.00	0.00	0.00	1.00	0.00	0.00	0.00
	overall	7.88	12.13	5.50	4.13	0.34	5.75	14.25	4.75	5.50	0.39
*I* = 40	0.00	1.00	0.00	1.00	0.00	-	1.00	0.00	1.00	0.00	-
	1	3.13	4.88	3.13	0.88	0.18	2.88	5.13	2.88	1.13	0.22
	2	7.25	18.75	5.00	1.5	0.08	4.25	21.75	3.63	3.13	0.14
	3	0.38	2.63	0.38	0.63	0.24	0.13	2.88	0.13	0.88	0.30
	4	0.38	1.63	0.00	0.00	0.00	0.13	1.88	0.00	0.00	0.00
	overall	12.13	27.88	9.50	3.00	0.11	8.38	31.63	7.63	5.13	0.160

**Table 4:** Impact of the objective function: waiting time and tardiness (absolute and weighted values) for operated and still waiting patients.

		DAS				RAS (Γ=4)				DAS		RAS (Γ=4)	
		Waiting Time		Weighted w.t.		Waiting Time		Weighted w.t.		Tardiness		Tardiness	
Instance	URG	wt_op	wt_sw	wwt_op	wwt_sw	wt_op	wt_sw	wwt_op	wwt_sw	trd_op	trd_sw	trd_op	trd_sw
*I* = 20	0	7.00	14.00	315.00	630.00	7.00	14.00	315.00	630.00	-	6.00	-	6.00
	1	52.65	29.79	631.75	357.50	66.38	31.92	796.50	383.00	37.83	20.67	41.67	22.00
	2	96.33	40.44	578.00	242.63	106.55	46.48	639.30	278.85	45.85	5.00	47.56	9.93
	3	227.00	179.66	454.00	359.31	-	181.75	-	363.50	47.00	31.75	-	34.00
	4	-	346.00	-	346.00	-	346.00	-	346.00	-	-	-	-
	overall	75.12	108.17	560.43	333.91	83.30	101.27	631.62	351.66	41.96	22.64	44.00	22.95
*I* = 40	0	13.00	-	585.00	-	13.00	-	-	-	5.00	-	5.00	-
	1	74.04	25.61	888.50	307.37	76.38	27.37	916.50	328.50	44.04	32.40	46.38	32.17
	2	74.31	42.47	445.88	254.80	87.10	44.80	522.58	268.77	24.60	3.07	31.38	8.42
	3	201.00	159.27	402.00	318.54	-	163.98	-	327.96	21.00	24.00	21.00	24.00
	4	-	342.94	-	342.94	-	342.94	-	342.94	-	-	-	-
	overall	79.43	68.06	563.70	276.20	76.61	70.47	659.55	289.19	28.46	14.03	32.68	14.96

**Table 5:** Robustness parameter analysis: number of operated patients.

Instance	Max	DAS	Γ=1	Γ=2	Γ=3	Γ=4	Γ=5	Γ=6	Γ=7	Γ=8
20−1	8	8	6	6	4	-	-	-	-	-
20−2	9	8	8	7	6	-	-	-	-	-
20−3	7	7	6	5	-	-	-	-	-	-
20−4	10	9	8	8	6	-	-	-	-	-
20−5	12	11	10	9	10	9	9	8	-	-
20−6	8	7	7	6	6	5	-	-	-	-
20−7	8	7	7	7	6	-	-	-	-	-
20−8	8	6	7	6	5	-	-	-	-	-
40−1	14	12	9	7	5	-	-	-	-	-
40−2	15	13	11	12	10	-	-	-	-	-
40−3	12	10	9	8	7	-	-	-	-	-
40−4	15	14	13	12	10	9	-	-	-	-
40−5	20	18	17	15	15	13	13	12	-	-
40−6	14	10	9	10	9	8	-	-	-	-
40−7	13	10	11	8	-	-	-	-	-	-
40−8	13	10	9	9	7	-	-	-	-	-

**Table 6:** Robustness parameter analysis: number of tardy patients operated (op) and still waiting (sw).

Instance	DAS		Γ = 1		Γ = 2		Γ = 3		Γ = 4		Γ = 5		Γ = 6		Γ = 7		Γ = 8	
	**op**	**sw**	**op**	**sw**	**op**	**sw**	**op**	**sw**	**op**	**sw**	**op**	**sw**	**op**	**sw**	**op**	**sw**	**op**	**sw**
20−1	5	5	4	6	3	7	4	7	-	-	-	-	-	-	-	-	-	-
20−2	5	4	5	5	4	5	5	5	-	-	-	-	-	-	-	-	-	-
20−3	5	5	4	6	-	-	-	-	-	-	-	-	-	-	-	-	-	-
20−4	7	2	7	3	6	4	5	5	-	-	-	-	-	-	-	-	-	-
20−5	7	2	7	2	7	2	6	3	6	3	6	4	6	3	-	-	-	-
20−6	5	5	5	5	4	5	4	6	-	-	-	-	-	-	-	-	-	-
20−7	5	5	5	6	5	6	4	6	5	6	-	-	-	-	-	-	-	-
20−8	5	5	5	6	-	-	-	-	-	-	-	-	-	-	-	-	-	-
40−1	8	5	7	6	6	7	5	8	-	-	-	-	-	-	-	-	-	-
40−2	11	2	10	3	8	4	9	4	-	-	-	-	-	-	-	-	-	-
40−3	10	3	8	5	8	5	7	6	-	-	-	-	-	-	-	-	-	-
40−4	11	0	12	1	12	1	10	3	9	4	-	-	-	-	-	-	-	-
40−5	11	1	12	1	11	1	11	1	10	1	12	1	11	1	11	1	12	1
40−6	9	4	8	5	7	5	7	5	7	6	-	-	-	-	-	-	-	-
40−7	9	4	8	5	8	5	7	6	-	-	-	-	-	-	-	-	-	-
40−8	8	5	8	5	7	6	-	-	-	-	-	-	-	-	-	-	-	-

**Table 7:** Computational times in seconds.

		DAS	Γ=1	Γ=2	Γ=3	Γ=4	Γ=5	Γ=6	Γ=7	Γ=8
20 patients	average	0.02	0.05	0.10	0.21	0.11	0.19	0.18	0.17	0.09
	max	0.03	0.08	0.20	1.14	0.34	0.73	0.59	0.56	0.37
40 patients	average	0.15	6.80	2.06	11.38	102.73	107.67	250.81	73.85	172.00
	max	0.82	52.59	10.32	42.37	613.86	645.20	1678.96	521.78	1334.47

**Table 8:** Percentage gap and computational time for the large instances, average values with respect to surgery time sets.

Instance Size		DAS		Γ=1		Γ=2		Γ=3		Γ=4		Γ=5		Γ=6		Γ=7		Γ=8	
80 patients	3 blocks																		
	average	ag	0.20	ag	1.85	ag	18.24	ag	150.34	0.21	1478.21	0.22	1114.95	0.25	1615.20	0.23	1250.34	0.22	962.83
	max	ag	1.13	ag	10.10	ag	95.68	ag	918.61	1.02	TL	1.05	TL	1.35	TL	1.17	TL	1.05	TL
	5 blocks																		
	average	ag	12.42	0.16	2006.67	0.57	3614.22	1.72	5322.70	1.87	5518.19	1.81	4004.13	1.83	4587.68	1.61	4271.28	1.49	3286.89
	max	ag	66.58	0.39	TL	2.06	TL	6.84	TL	7.80	TL	6.99	TL	6.68	TL	5.87	TL	5.26	TL
120 patients	3 blocks																		
	average	ag	0.18	ag	7.24	ag	37.41	ag	1495.08	0.18	1480.65	0.25	2058.85	0.27	1928.32	0.20	1618.33	0.18	1226.67
	max	ag	0.45	ag	22.82	ag	98.08	ag	5792.23	0.78	TL	0.81	TL	1.14	TL	0.93	TL	0.75	TL
	5 blocks																		
	average	ag	8.30	0.34	3581.94	0.84	6634.15	2.63	TL	2.67	TL	2.48	6638.56	2.23	TL	1.99	7034.86	1.71	TL
	max	ag	55.61	1.16	TL	2.45	TL	5.29	TL	6.21	TL	5.72	TL	4.86	TL	4.38	TL	3.89	TL
140 patients	3 blocks																		
	average	ag	0.19	ag	10.03	ag	35.55	0.17	1983.57	0.22	1891.15	0.20	1831.85	0.22	2014.69	0.15	1843.31	0.15	1103.61
	max	ag	0.40	ag	36.47	ag	93.00	0.61	TL	1.03	TL	0.91	TL	0.65	TL	0.42	TL	0.50	TL
	5 blocks																		
	average	ag	422.21	0.24	4393.92	0.83	TL	2.10	TL	2.04	7187.16	1.79	6933.17	1.68	6429.03	1.44	6373.13	1.29	6309.93
	max	0.12	3272.88	0.78	TL	1.21	TL	4.82	TL	5.33	TL	5.01	TL	4.52	TL	3.97	TL	3.64	TL

## Data Availability

Instance data are available on request from the authors.

## Appendix A

### Appendix A.1. Linearization of the Capacity Constraint of the Robust Model

For each block *j*, a subset $S_{j}$ of patients, who require their maximum surgery time ${\bar{t}}_{i}+{\hat{t}}_{i}$, such that $|S_{j}|=\Gamma$, is chosen among the patients assigned to the block. Among all the possible subsets, the one having the worst impact on the capacity constraint is selected, and the solution is guaranteed to be feasible even with respect to such subset:

(A1) $$\sum_{i\in I}{\bar{t}}_{i}x_{ij}+\max_{S_{j}\subset I:\left|S_{j}\right|=\Gamma }\left\{\sum_{i\in S_{j}}{\hat{t}}_{i}x_{ij}\right\}\leq \gamma_{j}\;\forall j\in J$$

The value $\max_{S_{j}\subset I:\left|S_{j}\right|=\Gamma }\left\{\sum_{i\in S_{j}}{\hat{t}}_{i}x_{ij}\right\}$ can be computed for each block *j* solving the following linear programming problem:

(A2) $$\begin{array}{ccc} \beta^{j}= & \max\left(\sum_{i\in I}{\hat{t}}_{i}x_{ij}\right)z_{i} & \end{array}$$

(A3) $$\begin{array}{ccc} \; & \sum_{i\in I}z_{i}\leq \Gamma & \end{array}$$

(A4) $$\begin{array}{ccc} \; & z_{i}\leq 1 & \;\forall i\in I \end{array}$$

(A5) $$\begin{array}{ccc} \; & z_{i}\geq 0 & \;\forall i\in I \end{array}$$

Let denote with $\zeta^{j}$ the dual variables associated with constraints (A3) and with $\pi_{i}^{j}$ the dual variables associated with constraints (A4). The dual of $\left(\beta^{j}\right)$ can be formulated as follows:

(A6) $$\begin{array}{c} \min\;\Gamma \zeta^{j}+\sum_{i\in I}\pi_{i}^{j} \end{array}$$

(A7) $$\begin{array}{cccc} \zeta^{j}+\pi_{i}^{j} & \geq {\hat{t}}_{i}x_{ij} & \; & \forall i\in I \end{array}$$

(A8) $$\begin{array}{c} \zeta^{j},\pi_{i}^{j}\geq 0 \end{array}$$

The optimal values of the objective functions (A2) and (A6) coincide. Thus, constraints (A1) can be linearized, by replacing them with:

(A9) $$\begin{array}{cccc} \sum_{i\in I}{\bar{t}}_{i}x_{ij}+\Gamma \zeta^{j}+\sum_{i\in I}\pi_{i}^{j} & \leq \gamma_{j} & \; & \forall j\in J \end{array}$$

(A10) $$\begin{array}{cccc} \zeta^{j}+\pi_{i}^{j} & \geq {\hat{t}}_{i}x_{ij} & \; & \;\forall j\in J\;\forall i\in I \end{array}$$

(A11) $$\begin{array}{c} \zeta^{j},\pi_{i}^{j}\geq 0 \end{array}$$

Therefore, the resulting RAS model formulation is as follows:

$$\begin{array}{ccc} \; & \min\sum_{i\in I}\left\{\sum_{j\in J}p_{ij}x_{ij}+q_{i}\left(1-\sum_{j\in J}x_{ij}\right)\right\} & \\ \; & \sum_{j\in J}x_{ij}\leq 1 & \forall i\in I\\ \; & \sum_{i\in I}{\bar{t}}_{i}x_{ij}+\Gamma \zeta^{j}+\sum_{i\in I}\pi_{i}^{j}\leq \gamma_{j} & \forall j\in J\\ \; & \zeta^{j}+\pi_{i}^{j}\geq {\hat{t}}_{i}x_{ij} & \forall j\in J,\forall i\in I\\ \; & \zeta^{j},\pi_{i}^{j}\geq 0 & \forall j\in J,\forall i\in I\\ \; & x_{ij}\in \left\{0,1\right\} & \forall j\in J,\forall i\in I \end{array}$$

### Appendix A.2. Appendix—Detailed Tables

Detailed tables are reported in this appendix.

**Table A1:** Detailed number of (#) operated and still waiting patients, waiting time and tardiness, for the 20 patient instances.

		DAS										RAS (Γ=4)									
		# Patients		# Tardy p.		Operated p.		Still Waiting p.		Tardiness		# Patients		# Tardy p.		Operated p.		Still Waiting		Tardiness	
Instance	URG	op	sw	op_t	sw_t	wt_op	wwt_op	wt_sw	wwt_sw	trd_op	trd_sw	op	sw	op_t	sw_t	wt_op	wwt_op	wt_sw	wwt_sw	trd_op	trd_sw
	0	0	1	0	1	-	-	14.00	630.00	-	6.00	0	1	0	1	-	-	14.00	630.00		6.00
	1	2	4	1	2	47.00	564.00	36.00	432.00	45.00	23.00	1	5	1	2	75.00	900.00	34.00	408.00	45.00	23.00
20−1	2	6	2	4	0	82.33	494.00	35.00	210.00	40.50	-	3	5	3	2	114.33	686.00	47.60	285.60	54.33	5.50
	3	0	4	0	2	-	-	181.75	363.50	-	34.00	0	4	0	2	-	-	181.75	363.50		34.00
	4	0	1	0	0	-	-	346.00	346.00	-	-	0	1	0	0	-	-	346.00	346.00		
	overall	8	12	5	5	73.50	511.50	108.42	381.50	41.40	24.00	4	16	4	7	104.50	739.50	93.44	368.63	52.00	18.71
	0	1	0	0	0	7.00	315.00	-	-	-	-	1	0	0	0	7.00	315.00	-	-	-	-
	1	3	3	2	1	49.67	596.00	28.00	336.00	35.00	16.00	1	5	1	2	75.00	900.00	34.00	408.00	45.00	23.00
20−2	2	4	4	3	1	100.50	603.00	43.00	258.00	54.33	5.00	4	4	4	1	101.00	606.00	43.00	258.00	41.00	5.00
	3	0	4	0	2	-	-	181.75	363.50	-	34.00	0	4	0	2	-	-	181.75	363.50	-	34.00
	4	0	1	0	0	-	-	346.00	346.00	-	-	0	1	0	0	-	-	346.00	346.00	-	-
	overall	8	12	5	4	69.75	564.38	110.75	320.00	46.60	22.25	6	14	5	5	81.00	606.50	101.07	348.00	41.80	23.80
	0	1	0	0	0	7.00	315.00	-	-	-	-	1	0	0	0	7.00	315.00	-	-	-	-
	1	2	4	1	2	48.00	576.00	36.00	432.00	45.00	23.00	1	5	1	2	77.00	924.00	34.00	408.00	47.00	23.00
20−3	2	4	4	4	1	101.00	606.00	43.00	258.00	41.00	5.00	3	5	3	2	114.33	686.00	47.60	285.60	54.33	5.50
	3	0	4	0	2	-	-	181.75	363.50	-	34.00	0	4	0	2	-	-	181.75	363.50	-	34.00
	4	0	1	0	0	-	-	346.00	346.00	-	-	0	1	0	0	-	-	346.00	346.00	-	-
	overall	7	13	5	5	72.43	555.86	106.85	350.77	41.80	23.28	5	15	4	6	85.40	659.40	98.73	351.20	52.50	20.83
	0	1	0	0	0	7.00	315.00	-	-	-	-	1	0	0	0	7.00	315.00	-	-	-	-
	1	3	3	3	0	56.33	676.00	21.33	256.00	26.33	-	3	3	3	0	56.33	676.00	21.33	256.00	26.33	-
20−4	2	4	4	3	1	100.50	603.00	43.00	258.00	54.33	5.00	2	6	2	3	131.00	786.00	54.33	326.00	71.00	13.00
	3	1	3	1	1	227.00	454.00	165.00	330.00	47.00	16.00	0	4	0	2	-	-	181.75	363.50	-	34.00
	4	0	1	0	0	-	-	346.00	346.00	-	-	0	1	0	0	-	-	346.00	346.00	-	-
	overall	9	11	7	2	89.44	578.78	97.91	285.09	41.29	10.50	6	14	5	5	73.00	652.50	104.50	323.14	44.20	21.40
	0	1	0	0	0	7.00	315.00	-	-	-	-	1	0	0	0	7.00	315.00	-	-	-	-
	1	4	2	3	0	47.50	570.00	19.00	228.00	26.33	-	3	3	2	1	49.67	596.00	28.00	336.00	35.00	16.00
20−5	2	6	2	4	0	83.83	503.00	30.50	183.00	40.50	-	5	3	4	0	92.40	554.40	35.67	214.00	40.50	-
	3	0	4	0	2	-	-	181.75	363.50	-	34.00	0	4	0	2	-	-	181.75	363.50	-	34.00
	4	0	1	0	0	-	-	346.00	346.00	-	-	0	1	0	0	-	-	346.00	346.00	-	-
	overall	11	9	7	2	63.64	510.27	130.22	291.33	34.43	34.00	9	11	6	3	68.67	541.67	114.91	313.64	38.67	28.00
	0	0	1	0	1	-	-	14.00	630.00	-	6.00	1	0	0	0	7.00	315.00	-	-	-	-
	1	3	3	2	1	49.67	596.00	28.00	336.00	35.00	16.00	1	5	1	2	75.00	900.00	34.00	408.00	45.00	23.00
20−6	2	4	4	3	1	100.00	600.00	43.00	258.00	53.67	5.00	3	5	3	2	98.33	590.00	57.60	345.60	38.33	30.50
	3	0	4	0	2	-	-	181.75	363.50	-	34.00	0	4	0	2	-	-	181.75	363.50	-	34.00
	4	0	1	0	0	-	-	346.00	346.00	-	-	0	1	0	0	-	-	346.00	346.00	-	-
	overall	7	13	5	5	78.43	598.29	103.31	343.85	46.20	19.00	5	15	4	6	75.40	597.00	102.07	371.20	40.00	29.17
	0	1	0	0	0	7.00	315.00	-	-	-	-	0	1	0	1	-	-	14.00	630.00	-	6.00
	1	2	4	1	2	48.00	576.00	36.00	432.00	45.00	23.00	2	4	1	2	48.00	576.00	36.00	432.00	45.00	23.00
20−7	2	4	4	4	1	101.00	606.00	43.00	258.00	41.00	5.00	4	4	4	1	100.50	603.00	43.00	258.00	40.50	5.00
	3	0	4	0	2	-	-	181.75	363.50	-	34.00	0	4	0	2	-	-	181.75	363.50	-	34.00
	4	0	1	0	0	-	-	346.00	346.00	-	-	0	1	0	0	-	-	346.00	346.00	-	-
	overall	7	13	5	5	72.43	555.86	106.85	350.77	41.80	23.80	6	14	5	6	83.00	594.00	100.21	370.71	41.40	20.83
	0	1	0	0	0	7.00	315.00	-	-	-	-	0	1	0	1	-	-	14.00	630.00	-	6.00
	1	1	5	1	2	75.00	900.00	34.00	408.00	45.00	23.00	1	5	1	2	75.00	900.00	34.00	408.00	45.00	23.00
20−8	2	4	4	4	1	101.50	609.00	43.00	258.00	41.50	5.00	4	4	4	1	100.50	603.00	43.00	258.00	40.50	5.00
	3	0	4	0	2	-	-	181.75	363.50	-	34.00	0	4	0	2	-	-	181.75	363.50	-	34.00
	4	0	1	0	0	-	-	346.00	346.00	-	-	0	1	0	0	-	-	346.00	346.00	-	-
	overall	6	14	5	5	81.33	608.50	101.07	348.00	42.20	23.80	5	15	5	6	95.40	662.40	95.27	366.80	41.40	20.83

**Table A2:** Detailed number of (#) operated and still waiting patients, waiting time and tardiness, for the 40 patient instances.

		DAS										RAS (Γ=4)									
		# Patients		# Tardy p.		Operated p.		Still Waiting p.		Tardiness		# Patients		# Tardy p.		Operated p.		Still Waiting		Tardiness	
Instance	URG	op	sw	op_t	sw_t	wt_op	wwt_op	wt_sw	wwt_sw	trd_op	trd_sw	op	sw	op_t	sw_t	wt_op	wwt_op	wt_sw	wwt_sw	trd_op	trd_sw
	0	1	0	1	0	13.00	585.00	-	-	5.00	-	1	0	1	0	13.00	585.00	-	-	5.00	-
	1	2	6	2	2	83.00	996.00	32.83	394.00	53.00	31.00	2	6	2	2	84.00	1008.00	32.83	394.00	54.00	31.00
40−1	2	9	17	5	2	62.56	375.33	44.41	266.47	24.80	3.50	2	24	2	5	104.00	624.00	47.46	284.75	44.00	12.00
	3	0	3	0	1	-	-	166.33	332.67	-	24.00	0	3	0	1	-	.	166.33	332.67	-	24.00
	4	0	2	0	0	-	-	342.50	342.50	-	-	0	2	0	0	-	.	342.50	342.50	-	-
	overall	12	28	8	5	61.83	496.25	76.29	306.32	29.38	18.60	5	35	5	8	77.80	769.80	72.00	310.89	40.20	18.25
	0	1	0	1	0	13.00	585.00	-	-	5.00	-	1	0	1	0	13.00	585.00	-	.	5.00	-
	1	4	4	4	0	69.50	834.00	18.75	225.00	39.50	-	3	5	3	1	71.67	860.00	28.60	343.20	41.67	38.00
40−2	2	8	18	5	1	71.00	426.00	41.50	249.00	24.00	2.00	6	20	5	2	79.33	476.00	42.65	255.90	25.20	3.50
	3	0	3	0	1	-	-	166.33	332.67	-	24.00	0	3	0	1	-	.	166.33	332.67	-	24.00
	4	0	2	0	0	-	-	342.50	342.50	-	-	0	2	0	0	-	.	342.50	342.50	-	-
	overall	13	27	10	2	66.08	563.77	74.30	261.67	28.30	13.00	10	30	9	4	70.40	602.10	72.67	283.90	28.44	17.25
	0	1	0	1	0	13.00	585.00	-	-	5.00	-	1	0	1	0	13.00	585.00	-	.	5.00	-
	1	3	5	3	1	71.00	852.00	28.60	343.20	41.00	38.00	3	5	3	1	71.67	860.00	28.60	343.20	41.67	38.00
40−3	2	5	21	5	2	85.20	511.20	43.14	258.86	25.20	3.50	3	23	3	4	98.67	592.00	45.48	272.87	38.67	6.75
	3	1	2	1	0	201.00	402.00	147.50	295.00	21.00	-	0	3	0	1	-	.	166.33	332.67	-	24.00
	4	0	2	0	0	-	-	342.50	342.50	-	-	0	2	0	0	-	.	342.50	342.50	-	-
	overall	10	30	10	3	85.30	609.90	67.63	280.90	27.50	15.00	7	33	7	6	74.86	705.86	71.91	293.18	35.14	14.83
	0	1	0	1	0	13.00	585.00	-	-	5.00	-	1	0	1	0	13.00	585.00	-	.	5.00	-
	1	4	4	4	0	69.00	828.00	18.75	225.00	39.00	-	4	4	4	0	69.00	828.00	18.75	225.00	39.00	-
40−4	2	7	19	5	0	77.00	462.00	41.00	246.00	24.00	-	4	22	4	3	91.50	549.00	44.23	265.36	31.50	4.67
	3	1	2	1	0	201.00	402.00	147.50	295.00	21.00	-	0	3	0	1	-	.	166.33	332.67	-	24.00
	4	1	1	0	0	343.00	343.00	339.00	339.00	-	-	0	2	0	0	-	.	342.50	342.50	-	-
	overall	14	26	11	0	98.00	562.57	57.23	250.12	27.45	-	9	31	9	4	72.78	677.00	72.00	271.65	31.89	9.50
	0	1	0	1	0	13.00	585.00	-	-	5.00	-	1	0	1	0	13.00	585.00	-	.	5.00	-
	1	4	4	4	0	69.50	834.00	18.75	225.00	39.50	-	4	4	4	0	69.00	828.00	18.75	225.00	39.00	-
40−5	2	11	15	5	1	61.09	366.55	41.73	250.40	23.60	2.00	6	20	4	1	80.67	484.00	42.05	252.30	31.00	2.00
	3	1	2	1	0	201.00	402.00	147.50	295.00	21.00	-	1	2	1	0	201.00	402.00	147.50	295.00	21.00	-
	4	1	1	0	0	336.00	336.00	346.00	346.00	-	-	1	1	0	0	336.00	336.00	346.00	346.00	-	-
	overall	18	22	11	1	83.33	482.83	61.00	254.18	27.45	2.00	13	27	10	1	100.77	579.92	57.67	254.89	30.60	2.00
	0	1	0	1	0	13.00	585.00	-	-	5.00	-	1	0	1	0	13.00	585.00	-	.	5.00	-
	1	3	5	3	1	76.33	916.00	25.80	309.60	46.33	24.00	3	5	3	1	77.67	932.00	25.80	309.60	47.67	24.00
40−6	2	6	20	5	2	79.00	474.00	42.65	255.90	24.80	3.50	4	22	3	4	80.00	480.00	46.05	276.27	30.67	11.75
	3	0	3	0	1	-	-	166.33	332.67	-	24.00	0	3	0	1	-	.	166.33	332.67	-	24.00
	4	0	2	0	0	-	-	342.50	342.50	-	-	0	2	0	0	-	.	342.50	342.50	-	-
	overall	10	30	9	4	71.60	617.70	72.20	278.30	29.28	13.75	8	32	7	6	70.75	662.63	72.69	290.91	34.29	15.83
	0	1	0	1	0	13.00	585.00	-	-	5.00	-	1	0	1	0	13.00	585.00	-	.	5.00	-
	1	3	5	3	1	71.00	852.00	28.60	343.20	41.00	38.00	2	6	2	2	84.00	1008.00	32.83	394.00	54.00	31.00
40−7	2	6	20	5	2	79.67	478.00	42.65	255.90	25.60	3.50	5	21	4	3	77.60	465.60	45.05	270.29	25.00	13.33
	3	0	3	0	1	-	-	166.33	332.67	-	24.00	0	3	0	1	-	.	166.33	332.67	-	24.00
	4	0	2	0	0	-	-	342.50	342.50	-	-	0	2	0	0	-	.	342.50	342.50	-	-
	overall	10	30	9	4	70.40	600.90	72.67	283.90	28.44	17.25	8	32	7	6	71.13	616.13	72.72	303.84	30.43	21.00
	0	1	0	1	0	13.00	585.00	-	-	5.00	-	1	0	1	0	13.00	585.00	-	.	5.00	-
	1	2	6	2	2	83.00	996.00	32.83	394.00	53.00	31.00	2	6	2	2	84.00	1008.00	32.83	394.00	54.00	31.00
40−8	2	6	20	5	2	79.00	474.00	42.65	255.90	24.80	3.50	4	22	4	3	85.00	510.00	45.41	272.45	25.00	13.33
	3	0	3	0	1	-	-	166.33	332.67	-	24.00	0	3	0	1	-	.	166.33	332.67	-	24.00
	4	1	1	0	0	336.00	336.00	346.00	346.00	-	-	0	2	0	0	-	.	342.50	342.50	-	-
	overall	10	30	8	5	98.90	575.70	63.17	294.20	29.38	18.60	7	33	7	6	74.43	663.00	72.12	304.27	30.43	21.00

**Table A3:** Percentage gap and computational time in seconds for the 80 patient instances.

**3 Blocks**	**DAS**		Γ=1		Γ=2		Γ=3		Γ=4		Γ=5		Γ=6		Γ=7		Γ=8	
**Instance**	**Gap**	**Time**	**Gap**	**Time**	**Gap**	**Time**	**Gap**	**Time**	**Gap**	**Time**	**Gap**	**Time**	**Gap**	**Time**	**Gap**	**Time**	**Gap**	**Time**
	**%**		**%**		**%**		**%**		**%**		**%**		**%**		**%**		**%**	
80−1	ag	0.05	ag	1.71	ag	0.66	ag	4.33	ag	2.48	ag	2.03	ag	1.59	ag	1.06	ag	0.76
80−2	ag	0.07	ag	0.44	ag	1.89	ag	81.77	ag	24.11	ag	11.76	ag	9.13	ag	5.12	ag	4.76
80−3	ag	0.02	ag	0.13	ag	0.65	ag	0.77	ag	0.65	ag	0.36	ag	0.95	ag	0.33	ag	0.20
80−4	ag	1.13	ag	1.45	ag	95.68	ag	918.61	1.02	TL	1.05	TL	ag	5711.39	ag	2803.27	ag	505.02
80−5	ag	0.09	ag	10.10	ag	46.41	ag	174.70	ag	4570.56	ag	1705.92	1.35	TL	1.17	TL	1.05	TL
80−6	ag	0.05	ag	0.54	ag	0.08	ag	0.16	ag	0.61	ag	0.52	ag	0.34	ag	0.58	ag	0.17
80−7	ag	0.05	ag	0.26	ag	0.09	ag	1.09	ag	20.59	ag	5.97	0.04	5.16	ag	2.92	ag	2.55
80−8	ag	0.11	ag	0.17	ag	0.43	ag	21.28	ag	19.08	ag	5.51	ag	5.42	ag	1.89	ag	1.60
average	ag	0.20	ag	1.85	ag	18.24	ag	150.34	0.21	1478.21	0.22	1114.95	0.25	1615.20	0.23	1250.34	0.22	962.83
max	ag	1.13	ag	10.10	ag	95.68	ag	918.61	1.02	TL	1.05	TL	1.35	TL	1.17	TL	1.05	TL
**5 Blocks**	**DAS**		Γ=1		Γ=2		Γ=3		Γ=4		Γ=5		Γ=6		Γ=7		Γ=8	
**Instance**	**Gap**	**Time**	**Gap**	**Time**	**Gap**	**Time**	**Gap**	**Time**	**Gap**	**Time**	**Gap**	**Time**	**Gap**	**Time**	**Gap**	**Time**	**Gap**	**Time**
	**%**		**%**		**%**		**%**		**%**		**%**		**%**		**%**		**%**	
80−1	ag	2.11	ag	195.93	ag	980.32	ag	1733.23	ag	883.74	ag	605.31	ag	293.38	ag	246.04	ag	163.64
80−2	ag	5.73	ag	1244.76	0.84	TL	2.21	TL	1.92	TL	1.55	TL	1.09	7147.55	0.80	7179.31	0.65	TL
80−3	ag	0.39	ag	55.76	0.38	7187.49	ag	2051.23	ag	1150.95	ag	167.61	ag	168.70	ag	128.74	ag	98.69
80−4	ag	22.05	0.30	7181.18	2.06	7186.28	6.84	TL	7.80	TL	6.99	TL	6.68	5745.76 *	5.87	6634.73 *	5.26	TL
80−5	ag	66.58	0.39	TL	0.86	5235.98 *	2.25	TL	3.14	TL	4.65	790.13 *	5.64	TL	5.21	TL	5.08	992.23 *
80−6	ag	1.36	ag	13.56	ag	941.52	0.51	TL	ag	7020.63	ag	3616.01	ag	4491.46	ag	2312.77	ag	813.17
80−7	ag	0.58	ag	170.10	ag	187.39	ag	2859.41	0.47	TL	ag	5597.01	ag	4479.43	ag	3293.56	ag	2664.78
80−8	ag	0.53	ag	4.53	ag	7.25	1.65	TL	1.32	6340.00 *	0.90	6881.90 *	0.83	TL	0.60	TL	0.49	TL
average	ag	12.42	0.16	2006.67	0.57	3614.22	1.72	5322.70	1.87	5518.19	1.81	4004.13	1.83	4587.68	1.61	4271.28	1.49	3286.89
max	ag	66.58	0.39	TL	2.06	TL	6.84	TL	7.80	TL	6.99	TL	6.68	TL	5.87	TL	5.26	TL

**Table A4:** Percentage gap and computational time in seconds for the 120 patients instances.

**3 Blocks**	**DAS**		Γ=1		Γ=2		Γ=3		Γ=4		Γ=5		Γ=6		Γ=7		Γ=8	
**Instance**	**Gap**	**Time**	**Gap**	**Time**	**Gap**	**Time**	**Gap**	**Time**	**Gap**	**Time**	**Gap**	**Time**	**Gap**	**Time**	**Gap**	**Time**	**Gap**	**Time**
	**%**		**%**		**%**		**%**		**%**		**%**		**%**		**%**		**%**	
120−1	ag	0.05	ag	2.12	ag	43.89	ag	358.42	ag	197.04	ag	150.19	ag	99.61	ag	54.76	ag	54.21
120−2	ag	0.31	ag	22.82	ag	5.37	ag	1625.98	ag	211.47	ag	78.50	ag	48.97	ag	31.27	ag	10.21
120−3	ag	0.07	ag	0.75	ag	51.25	ag	83.23	ag	52.47	ag	64.26	ag	44.87	ag	23.04	ag	2.78
120−4	ag	0.45	ag	5.61	ag	14.54	ag	5792.23	0.78	TL	0.81	TL	0.45	TL	ag	5227.71	ag	2449.56
120−5	ag	0.09	ag	12.20	ag	98.08	ag	1290.97	ag	1527.07	0.61	TL	1.14	TL	0.93	TL	0.75	TL
120−6	ag	0.19	ag	11.60	ag	81.35	ag	116.02	ag	178.74	ag	82.43	ag	66.48	ag	50.39	ag	14.90
120−7	ag	0.09	ag	1.77	ag	3.42	ag	72.58	ag	115.49	ag	76.87	ag	66.40	ag	39.22	ag	28.46
120−8	ag	0.15	ag	1.08	ag	1.39	ag	2621.18	ag	2375.38	ag	1643.39	ag	725.07	ag	332.66	ag	65.71
average	ag	0.18	ag	7.24	ag	37.41	ag	1495.08	0.18	1480.65	0.25	2058.85	0.27	1928.32	0.20	1618.33	0.18	1226.67
max	ag	0.45	ag	22.82	ag	98.08	ag	5792.23	0.78	TL	0.81	TL	1.14	TL	0.93	TL	0.75	TL
**5 Blocks**	**DAS**		Γ=1		Γ=2		Γ=3		Γ=4		Γ=5		Γ=6		Γ=7		Γ=8	
**Instance**	**Gap**	**Time**	**Gap**	**Time**	**Gap**	**Time**	**Gap**	**Time**	**Gap**	**Time**	**Gap**	**Time**	**Gap**	**Time**	**Gap**	**Time**	**Gap**	**Time**
	**%**		**%**		**%**		**%**		**%**		**%**		**%**		**%**		**%**	
120−1	ag	0.46	1.16	6409.59 *	2.45	TL	4.01	TL	2.19	TL	2.04	TL	1.80	TL	1.68	TL	1.19	TL
120−2	ag	7.64	0.44	TL	0.50	TL	2.05	TL	2.23	TL	1.88	TL	1.55	TL	1.27	TL	0.95	TL
120−3	ag	0.44	ag	227.65	1.43	TL	1.76	TL	1.47	TL	0.97	TL	0.77	TL	0.57	TL	0.50	TL
120−4	ag	55.61	0.52	TL	0.68	TL	5.29	TL	6.21	TL	5.72	TL	4.86	TL	4.38	TL	3.89	TL
120−5	ag	0.34	0.17	TL	0.55	TL	1.85	TL	2.87	TL	3.75	2795.42 *	4.19	TL	3.86	5965.84 *	3.50	TL
120−6	ag	0.59	ag	145.12	0.73	TL	1.62	TL	1.81	TL	1.53	TL	1.21	TL	1.03	TL	0.86	TL
120−7	ag	0.55	ag	50.56	0.27	TL	1.36	TL	1.88	TL	1.52	TL	1.32	TL	1.15	TL	0.94	TL
120−8	ag	0.75	ag	259.97	ag	2760.24	3.06	TL	2.71	TL	2.43	TL	2.12	TL	1.99	TL	1.88	TL
average	ag	8.30	0.34	3581.94	0.84	6634.15	2.63	TL	2.67	TL	2.48	6638.56	2.23	TL	1.99	7034.86	1.71	TL
max	ag	55.61	1.16	TL	2.45	TL	5.29	TL	6.21	TL	5.72	TL	4.86	TL	4.38	TL	3.89	TL

**Table A5:** Percentage gap and computational time in seconds for the 140 patients instances.

**3 Blocks**	**DAS**		Γ=1		Γ=2		Γ=3		Γ=4		Γ=5		Γ=6		Γ=7		Γ=8	
**Instance**	**Gap**	**Time**	**Gap**	**Time**	**Gap**	**Time**	**Gap**	**Time**	**Gap**	**Time**	**Gap**	**Time**	**Gap**	**Time**	**Gap**	**Time**	**Gap**	**Time**
	**%**		**%**		**%**		**%**		**%**		**%**		**%**		**%**		**%**	
140−1	ag	0.07	ag	36.47	ag	20.16	ag	283.55	ag	77.67	ag	35.58	ag	19.58	ag	11.82	ag	8.09
140−2	ag	0.13	ag	15.06	ag	51.87	ag	497.16	ag	460.48	ag	168.72	ag	138.32	ag	55.13	ag	61.78
140−3	ag	0.08	ag	1.15	ag	93.00	ag	16.94	ag	25.44	ag	5.73	ag	5.42	ag	2.60	ag	1.46
140−4	ag	0.32	ag	1.89	ag	67.19	0.61	TL	1.03	TL	0.91	TL	0.65	TL	0.26	TL	ag	1494.30
140−5	ag	0.37	ag	20.56	ag	20.97	ag	634.35	ag	471.84	ag	1664.47	0.54	TL	0.42	TL	0.50	TL
140−6	ag	0.11	ag	0.66	ag	14.16	ag	34.42	ag	14.03	ag	6.64	ag	3.59	ag	1.81	ag	1.15
140−7	ag	0.40	ag	0.87	ag	15.20	ag	26.98	ag	65.36	ag	46.07	ag	27.47	0.02	9.51	ag	3.03
140−10	ag	0.06	ag	3.58	ag	1.82	0.18	TL	ag	6826.82	ag	5540.05	ag	1548.02	ag	290.39	ag	71.50
average	ag	0.19	ag	10.03	ag	35.55	0.17	1983.57	0.22	1891.15	0.20	1831.85	0.22	2014.69	0.15	1843.31	0.15	1103.61
max	ag	0.40	ag	36.47	ag	93.00	0.61	TL	1.03	TL	0.91	TL	0.65	TL	0.42	TL	0.50	TL
**5 Blocks**	**DAS**		Γ=1		Γ=2		Γ=3		Γ=4		Γ=5		Γ=6		Γ=7		Γ=8	
**Instance**	**Gap**	**Time**	**Gap**	**Time**	**Gap**	**Time**	**Gap**	**Time**	**Gap**	**Time**	**Gap**	**Time**	**Gap**	**Time**	**Gap**	**Time**	**Gap**	**Time**
	**%**		**%**		**%**		**%**		**%**		**%**		**%**		**%**		**%**	
140−1	ag	0.27	0.78	TL	1.18	TL	2.27	TL	1.08	TL	0.76	TL	0.73	TL	0.64	TL	0.47	TL
140−2	ag	99.08	0.31	TL	0.86	TL	2.13	TL	1.95	TL	1.59	TL	1.21	TL	0.90	TL	0.72	TL
140−3	ag	0.56	ag	60.04	0.98	TL	0.93	TL	0.39	TL	ag	5152.33	ag	1119.23	ag	671.90	ag	166.43
140−4	ag	3.09	ag	34.44	1.21	TL	4.82	TL	5.33	TL	5.01	TL	4.52	TL	3.97	TL	3.64	TL
140−5	0.12	3272.88 *	0.24	TL	0.81	TL	1.72	TL	2.51	TL	2.83	TL	3.33	TL	3.11	TL	2.80	TL
140−6	ag	1.26	0.22	TL	0.66	TL	1.03	TL	1.18	TL	0.78	TL	0.69	TL	0.30	TL	0.34	TL
140−7	ag	0.30	ag	148.32	0.41	TL	1.18	TL	1.43	7184.21	1.13	TL	0.98	TL	0.81	TL	0.73	TL
140−10	ag	0.21	ag	6158.32	0.52	TL	2.70	TL	2.45	TL	2.15	TL	1.91	TL	1.70	TL	1.54	TL
average	ag	422.21	0.24	4393.92	0.83	TL	2.10	TL	2.04	7187.16	1.79	6933.17	1.68	6429.03	1.44	6373.13	1.29	6309.93
max	0.12	3272.88	0.78	TL	1.21	TL	4.82	TL	5.33	TL	5.01	TL	4.52	TL	3.97	TL	3.64	TL
